# *RASGRP1* targeted by H3K27me3 regulates myoblast proliferation and differentiation in mice and pigs: *RASGRP1* regulates myoblast proliferation and differentiation

**DOI:** 10.3724/abbs.2024011

**Published:** 2024-02-28

**Authors:** Liyao Xiao, Jiaxin Qiao, Yiyang Huang, Baohua Tan, Linjun Hong, Zicong Li, Gengyuan Cai, Zhenfang Wu, Enqin Zheng, Shanshan Wang, Ting Gu

**Affiliations:** 1 National Engineering Research Center for Breeding Swine Industry College of Animal Science South China Agricultural University Guangzhou 510000 China; 2 State Key Laboratory for Conservation and Utilization of Subtropical Agro-bioresources Guangzhou 510000 China; 3 Guangdong Provincial Laboratory of Lingnan Modern Agricultural Science and Technology Guangzhou 510000 China; 4 Guangdong Provincial Key Laboratory of Agro-animal Genomics and Molecular Breeding Guangzhou 510000 China; 5 Guangdong Wens Breeding Swine Technology Co. Ltd. Yunfu 527400 China; 6 College of Life Science Hubei University Wuhan 430000 China

**Keywords:** RASGRP1, H3K27me3, cell proliferation, myoblasts, skeletal muscle

## Abstract

Skeletal muscle is not only the largest organ in the body that is responsible for locomotion and exercise but also crucial for maintaining the body’s energy metabolism and endocrine secretion. The trimethylation of histone H3 lysine 27 (H3K27me3) is one of the most important histone modifications that participates in muscle development regulation by repressing the transcription of genes. Previous studies indicate that the *RASGRP1* gene is regulated by H3K27me3 in embryonic muscle development in pigs, but its function and regulatory role in myogenesis are still unclear. In this study, we verify the crucial role of H3K27me3 in *RASGRP1* regulation. The gain/loss function of *RASGRP1* in myogenesis regulation is performed using mouse myoblast C2C12 cells and primarily isolated porcine skeletal muscle satellite cells (PSCs). The results of qPCR, western blot analysis, EdU staining, CCK-8 assay and immunofluorescence staining show that overexpression of *RASGRP1* promotes cell proliferation and differentiation in both skeletal muscle cell models, while knockdown of *RASGRP1* leads to the opposite results. These findings indicate that *RASGRP1* plays an important regulatory role in myogenesis in both mice and pigs.

## Introduction

Skeletal muscle is not only the largest organ responsible for animal locomotion and exercise capacity but also crucial for maintaining the body’s energy metabolism and endocrine secretion [ 1– 3]. In livestock-producing fields, skeletal muscle is of particular importance, as meat is the main animal product, in addition to animal fur, milk and others. Increasing evidence indicates that epigenetic regulators participate in the development of skeletal muscle.

Epigenetic regulators such as DNA methylation, histone methylation, microRNAs (miRNAs) and long noncoding RNAs (lncRNAs) play vital roles in regulating myogenesis by affecting the transcription process or posttranscriptional modifications of RNAs [ 4– 7]. Among them, the trimethylation of histone H3 lysine 27 (H3K27me3) is a well-known repressive marker involved in various biological processes [ 8– 10]. H3K27me3 participates in the regulation of myogenic differentiation by silencing muscle-specific genes and cell cycle genes [ 11– 14]. The loss of H3K27me3 in the gene body, especially in the promoter of genes, repressed the differentiation of skeletal muscle cells [ 15– 17]. The methyltransferase of H3K27me3, named enhancer of zeste homolog 2 (EZH2), which is a subunit of polycomb repressive complex 2 (PRC2), can also directly or indirectly regulate the expressions of myogenic genes [ 18, 19]. During myogenic differentiation, the phosphorylated EZH2 enhancer induces a shift from H3K4me3 to H3K27me3 on the *Pax7* promoter to downregulate gene expression [20]. In contrast, the decrease in H3K27me3 is related to genes that are more actively transcribed [21] .

The *RASGRP1* gene is a member of the Ras gene family, which is highly expressed in T cells [22]. It has been reported that dysregulation of *RASGRP1* leads to the occurrence of cancers [ 23 – 28] and other diseases [ 29– 33] related to the body’s immunity [ 34 , 35]. Our previous study found that *RASGRP1* may regulate pig embryonic myogenesis, which was regulated by H3K27me3 through chromatin immunoprecipitation-sequencing (ChIP-seq) and RNA-sequencing of the longissimus dorsi muscle in Duroc pig embryos at gestation days 33 (E33), 65 (E65), and 90 (E90) [36]. However, the detailed function of this gene in regulating myogenesis is still unclear.

In this study, we first confirmed that the expression of the *RASGRP1* gene was regulated by H3K27me3 enrichment in its promoter during skeletal muscle development. Then, we studied the function of *RASGRP1* during cell proliferation and differentiation in both mouse C2C12 myoblasts and PSCs. Our results demonstrated that *RASGRP1* is regulated by H3K27me3 and promotes myogenesis in pigs and mice.

## Materials and Methods

### Animals

Pigs, including Duroc sows for embryonic skeletal muscle collection and newborn piglets for isolating porcine skeletal muscle satellite cells, were purchased from Guangdong Wen’s Foodstuffs Group (Yunfu, China). All animal experiments were conducted following the requirements for the Care and Use of Laboratory Animals by the Ethics Committee of the Laboratory Animal Center of South China Agricultural University, Guangzhou, China (Permit Number 2021F036, Permit Date 2 March 2021) [37].

### Cell culture

C2C12 cells were purchased from the Cell Bank of the Chinese Academy of Sciences (Shanghai, China). Dulbecco’s modified Eagle’s medium (DMEM) high glucose (Gibco, Grand Island, USA) and 10% fetal bovine serum (FBS; Gibco) was used for cell culture. For cell differentiation, DMEM high glucose with 2% horse serum (Gibco) was used. The cells were cultured with 5% CO _2_ at 37°C.

### Isolation and culture of porcine skeletal muscle satellite cells

Porcine skeletal muscle satellite cells (PSCs) were isolated from one-week-old piglets and cultivated according to protocols described in previous studies [ 38, 39]. The tissues were digested with 2 mg/mL type II collagenase (Sigma-Aldrich, Darmstadt, Germany) and then placed in a 37°C incubator for 2.5 h. Then, the digestion was stopped with an equal volume of RPMI 1640 medium (Gibco) containing 1% penicillin-streptomycin (P-S; Gibco). Then, 100, 200, and 400-mesh sieves were used to filter the cell suspension. Cell proliferation medium was prepared according to a previous study and used to resuspend cells [39]. Differential adhesion was used to obtain purified cells after 2 h of culture. When cells reached 70%‒80% confluence, differentiation medium consisting of DMEM high glucose with 2% horse serum and 1% P-S was used to induce cell differentiation.

### Plasmid construction, small interfering RNA synthesis, and transfection

The coding sequence of the porcine *RASGRP1* gene was cloned *in vitro* by PCR amplification and connected to the linear pcDNA3.1(+) vector (Sangon, Shanghai, China) to produce the pcDNA3.1- *RASGRP1* plasmid. The primers for the *RASGRP1* CD sequence were as follows: forward primer 5′-TACCGAGCTCGGATCCATGGGCACCCTGGGCAAG-3′ and reverse primer 5′-GATATCTGCAGAATTCCTAAGAACAGTCACCGTGCTCCATC-3′. Small interfering RNAs (siRNAs) against pig *RASGRP1* and mouse *RASGRP1* genes were designed and synthesized by GenePharma (Shanghai, China). The siRNA sequences were as follows: Sus-siRNA- *RASGRP1* (sense 5′-CCCAGUGGGUUCAACUCAUTT, antisense 5′-AUGAGUUGAACCCACUGGGTT-3′), Mus-siRNA- *RASGRP1* (sense 5′-GGACCUCAUAUCCCUGUAUTT-3′, antisense 5′-AUACAGGGAUAUGAGGUCCTT-3′), and siRNA negative control (sense: 5′-UUCUCCGAACGUGUCACGUTT-3′, antisense: 5′-ACGUGACACGUUCGGAGAATT-3′). Lipofectamine 3000 (Invitrogen, Carlsbad, USA) was used to conduct cell transfection following the manufacturer’s protocol.

### Quantitative real-time polymerase chain reaction (qPCR)

Total RNA kit ll (Omega Biotek, Norcross, USA) was used to harvest the total RNA following the manufacturer’s protocol. cDNA was prepared using a PrimeScript RT reagent kit (TaKaRa, Tokyo, Japan) and used to perform qPCR in a Quant Studio 7 Flex system (Thermo Fisher, Scientific, Waltham, USA). All primers used are presented in Supplementary Table S1.

### Chromatin immunoprecipitation (ChIP)

ChIP was conducted according to the protocol in a previous study [40]. Briefly, micrococcal nuclease was used to fragment chromatin. After that, the chromatin fragments were incubated with magnetic beads (Biogle, Wuxi, China). The DNA-bead complex was immunoprecipitated with anti-H3K27me3 antibody (Millipore, Billerica, USA) or negative control IgG. The primers for the promoter of the *RASGRP1* gene used for ChIP-qPCR were as follows: forward primer 5′-CTCTCCGAATTCCCCATTGTG-3′ and reverse primer 5′-AAATCAGAGCTGCATCCAC-3′.

### Western blot analysis

The concentration of protein harvested from cells by RIPA buffer with 1% PMSF (Beyotime, Shanghai, China) was determined using a BCA Assay Kit (Thermo Fisher Scientific). Western blot analysis was performed following a previous study [39]. The antibodies used were as follows: Ki67 (ab16667; 1:1000; Abcam, Cambridge, UK), CDK2 (PA1547; 1:1000; Boster, Pleasanton, USA), MyoG (sc-12732; 1:500; Santa Cruz Biotechnology, Santa Cruz, USA), MyoD (sc-377460; 1:500; Santa Cruz Biotechnology), MyHC (sc-376157; 1:1000; Santa Cruz Biotechnology), β-Tubulin (GB11017; 1:1000; Servicebio, Wuhan, China), goat anti-mouse IgG (A0216; 1:3000; Beyotime) and goat anti-rabbit IgG (A0208; 1:3000; Beyotime).

### Cell proliferation and cell cycle assays

For flow cytometry analysis, C2C12 cells stored at ‒20°C were fixed in 70% (v/v) ethanol overnight and treated at 4°C in 50 mg/mL propidium iodide for 30 min. Cell cycle detection was conducted in accordance with a previous study [39] by using a flow cytometer (Becton Dickinson, Franklin Lakes, USA). The cells were transfected with *RASGRP1* siRNA or overexpression vectors when they reached approximately 50% confluence. CCK-8 assays (Yeasen, Shanghai, China) were conducted following the manufacturer’s protocol after transfection. EdU staining was performed following the manufacturer’s protocol (RiboBio, Guangzhou, China). Cells were to fixed and permeabilized separately with 4% paraformaldehyde and 0.5% Triton X-100, respectively. DAPI was used to stain cell nuclei. All images were captured with the TE2000U fluorescence microscope imaging system (Nikon, Tokyo, Japan).

### Immunofluorescence staining

Cells seeded in a 24-well plate were transfected with *RASGRP1* siRNA or overexpression vectors when they reached approximately 50%–70% confluence. Then, 4% paraformaldehyde and 0.5% Triton X-100 were separately used to fix and permeabilize the cells for 30 min three days after transfection. Then, the cells were blocked in QuickBlock Buffer (Beyotime) at 37°C for 2 h and treated with the anti-MyHC antibody (sc-376157; 1:200; Santa Cruz Biotechnology) at 4°C overnight. Then, the cells were incubated with Alexa Fluor 488 fluorescent antibody (1:500; Thermo Fisher Scientific) after wash with PBS (Servicebio). DAPI was used to stain cell nuclei. All images were captured with the TE2000U fluorescence microscope imaging system.

### Statistical analysis

Data are presented as the mean±standard error of the mean (SEM). The comparative threshold cycle (2 ^–ΔΔct^) method was used to quantify the relative mRNA levels [ 38, 41]. Statistical analysis between different groups was performed by using two-tailed Student’s *t*-test or one-way analysis of variance (ANOVA) in SPSS software (version 22.0). ImageJ software was used for the quantification of positively-stained cells and the visualization of western blots. All experiments were performed in triplicate. *P*<0.05 indicated significant difference.

## Results

### *RASGRP1* is targeted by H3k27me3

**Figure 1:**
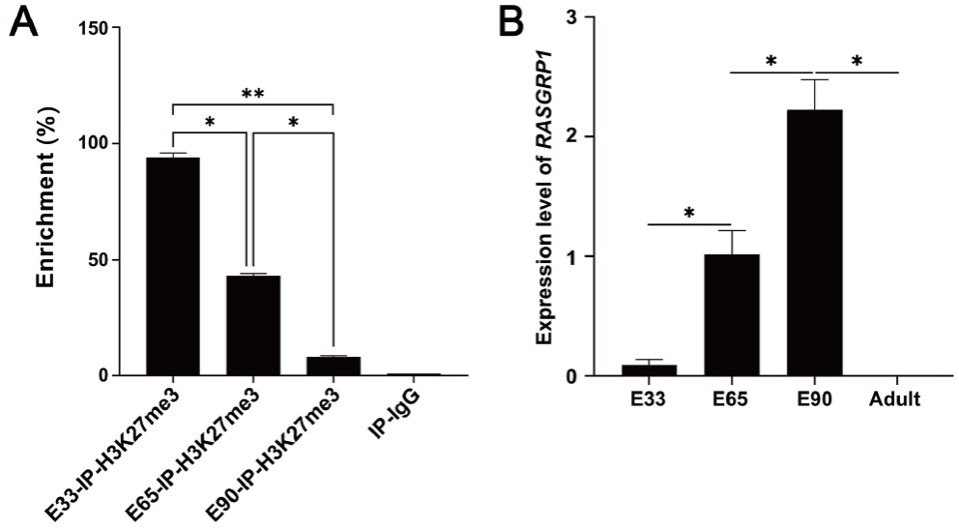
Figure 1 *RASGRP1* is targeted by H3K27me3 and upregulated in the longissimus dorsi muscle of Duroc pig embryos at gestation days 33 (E33), 65 (E65), and 90 (E90) (A) The enrichment of H3K27me3 in the RASGRP1 promoter at E33, E65, and E90. (B) The expression level of RASGRP1 in pig skeletal muscle at E33, E65, E90 and the adult period. β-Actin was used as a control. *P<0.05, **P<0.01.

We collected longissimus dorsi muscles on gestation days 33, 65, and 90, which represent three critical developmental time points for embryonic myogenesis [ 42– 45]. ChIP-qPCR assay in skeletal muscles at E33, E65, and E90 showed that the enrichment of H3K27me3 in the *RASGRP1* promoter was highest at E33 and lowest at E90 ( Figure 1A). The mRNA expression of the *RASGRP1* gene significantly increased during embryonic development ( Figure 1B and Supplementary Figure S1), while its expression was pretty low in adult skeletal muscle. These results indicated that during embryonic porcine myogenesis, increased *RASGRP1* expression might be specifically regulated by H3K27me3.

### *RASGRP1* promotes the proliferation of C2C12 cells

**Figure 2:**
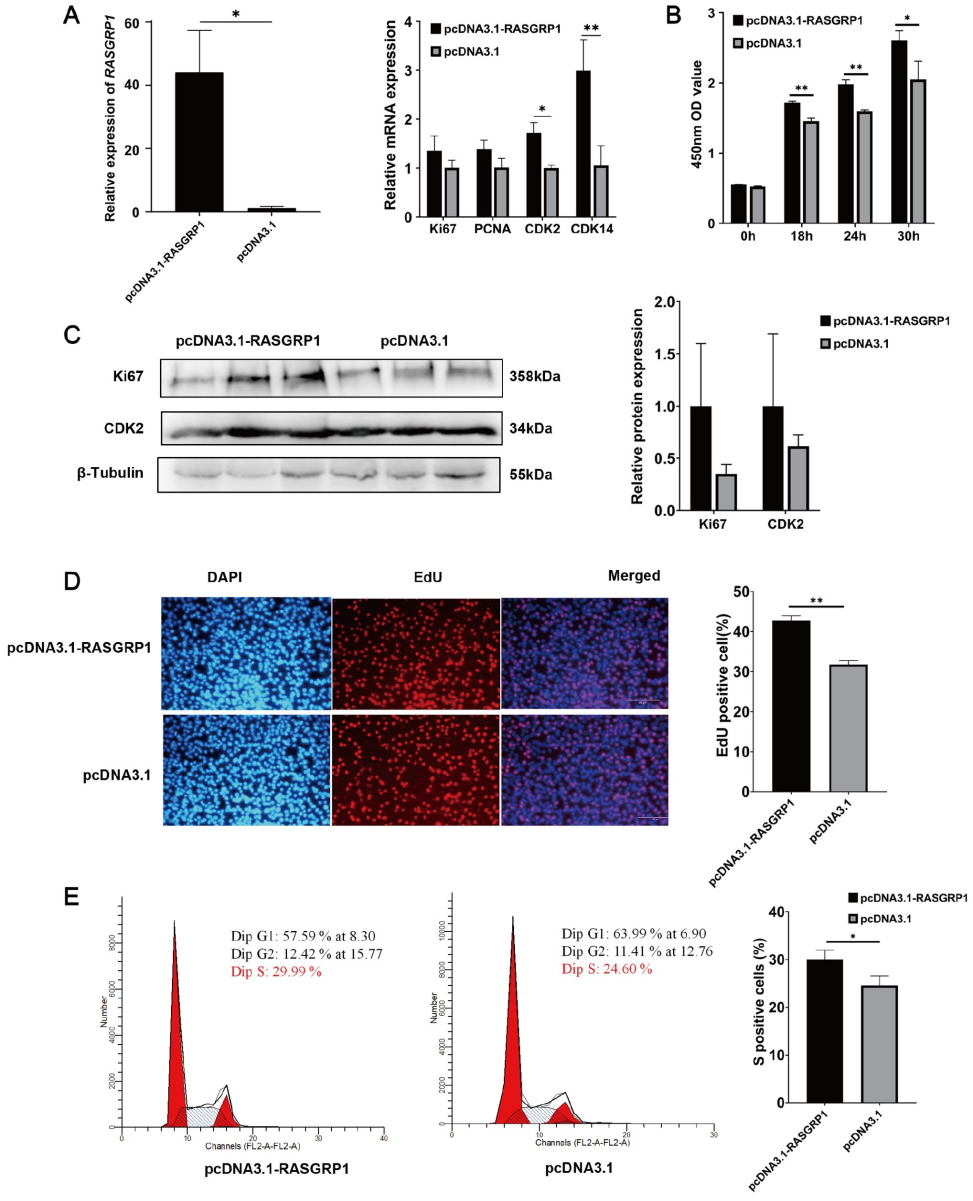
Figure 2 *RASGRP1* overexpression promotes the proliferation of C2C12 cells (A) The relative expression levels of RASGRP1 and proliferation marker genes after RASGRP1 overexpression. (B) Detection of C2C12 cell viability by CCK-8 assay at 18 h, 24 h and 30 h after RASGRP1 overexpression. (C) The relative protein levels of the Ki67 and CDK2 genes after RASGRP1 overexpression. (D) EdU staining after RASGRP1 overexpression in C2C12 cells. Scale bar: 100 μm. (E) Detection of the number of S-phase cells by flow cytometry after RASGRP1 overexpression. *P<0.05, **P<0.01.

To investigate the role of the *RASGRP1* gene in C2C12 cell proliferation, we performed gain- and loss-of-function analyses of the *RASGRP1* gene. We overexpressed the *RASGRP1* gene in C2C12 cells, and the qPCR results demonstrated that the relative expression of the *RASGRP1* gene and the proliferation marker genes *CDK2* and *CDK14* were significantly increased ( Figure 2A). CCK-8 assay demonstrated that *RASGRP1* overexpression also significantly promoted the proliferation of C2C12 cells ( Figure 2B). The relative protein expressions of the proliferation marker genes *Ki67* and *CDK2* were also increased, according to western blot analysis ( Figure 2C). EdU staining revealed that *RASGRP1* overexpression dramatically increased the percentage of EdU-positive (EdU ^+^) cells ( Figure 2D). In addition, flow cytometry analysis showed that there were 5.33% more S-phase cells among the cells overexpressing *RASGRP1* than in the pcDNA3.1(+) control group ( Figure 2E). All the above results indicated that *RASGRP1* promoted C2C12 cell proliferation.

**Figure 3:**
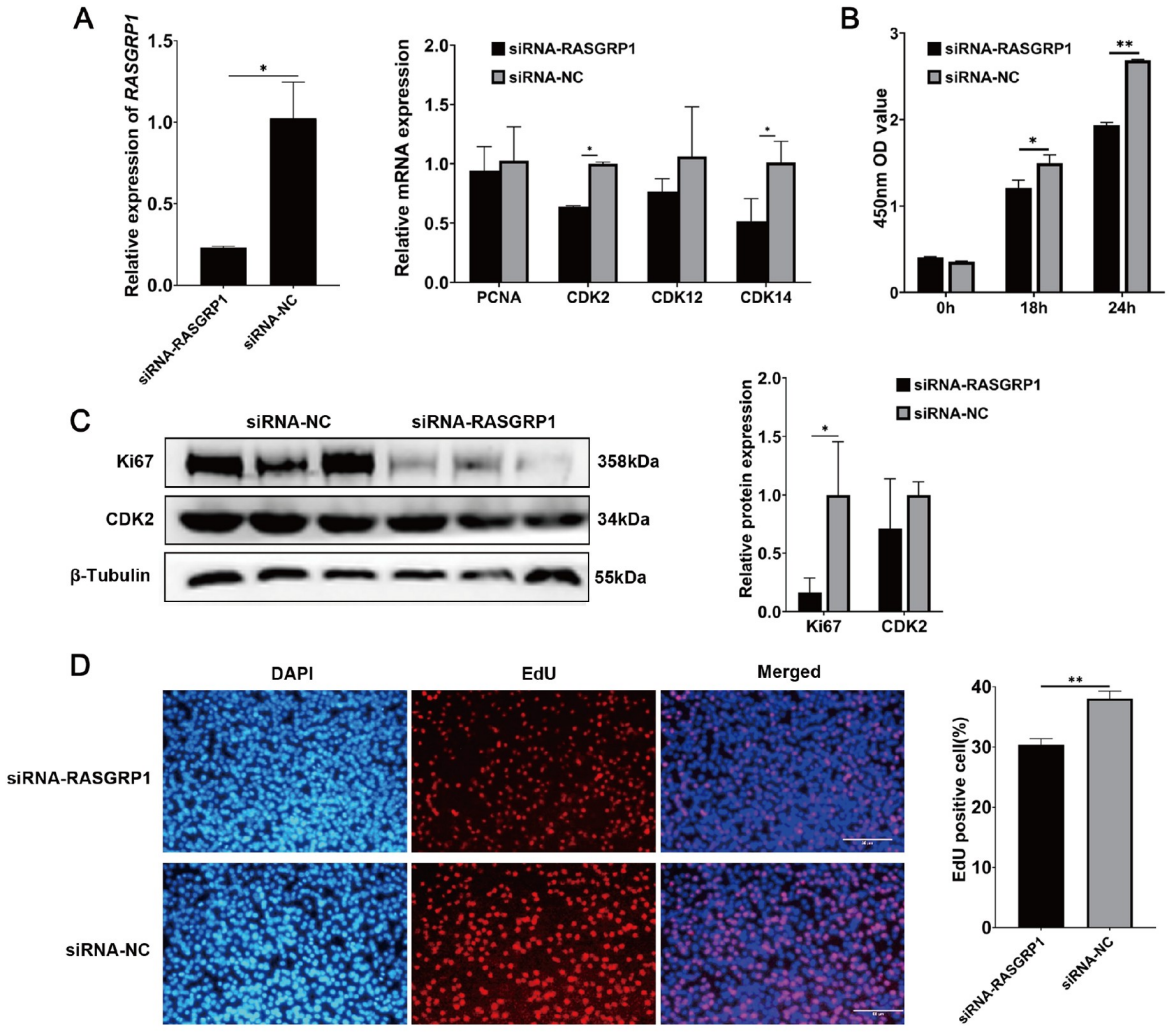
Figure 3 *RASGRP1* knockdown inhibits the proliferation of C2C12 cells (A) The relative expression levels of the RASGRP1 gene and proliferation marker genes after RASGRP1 knockdown. (B) Detection of C2C12 cell viability by CCK-8 assay at 18 h and 24 h after RASGRP1 knockdown. (C) The relative protein expression levels of the Ki67 and CDK2 genes after RASGRP1 knockdown. (D) EdU staining after RASGRP1 knockdown in C2C12 cells. Scale bar: 100 μm. *P<0.05, **P<0.01.

To verify the effect of *RASGRP1* overexpression on C2C12 cell proliferation, we then knocked down *RASGRP1* by siRNA in C2C12 cells and detected its effect on C2C12 cell proliferation. The qPCR results showed that the relative expression of *RASGRP1* as well as *CDK2* and *CDK14* was significantly reduced after *RASGRP1* knockdown ( Figure 3A). CCK-8 assay demonstrated that *RASGRP1* knockdown also dramatically reduced the proliferation activity of C2C12 cells ( Figure 3B). The relative protein expression of the *Ki67* gene was markedly decreased ( Figure 3C). EdU staining revealed that *RASGRP1* knockdown dramatically reduced the percentage of EdU ^+^ cells ( Figure 3D). These results indicated that *RASGRP1* knockdown inhibited the proliferation of C2C12 cells, which was opposite to the positive effect of *RASGRP1* overexpression on the proliferation of C2C12 cells.

### *RASGRP1* promotes the differentiation of C2C12 cells

**Figure 4:**
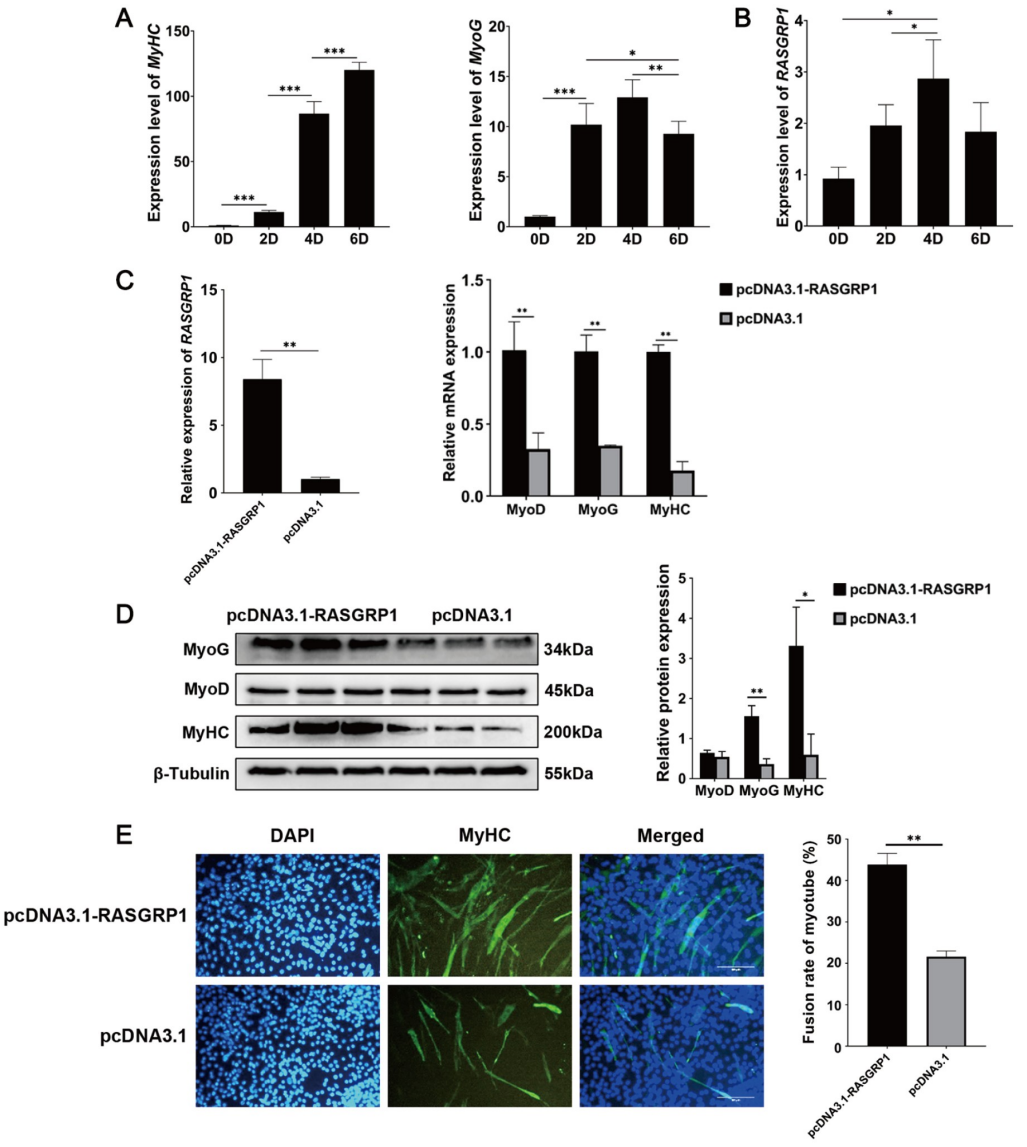
Figure 4 *RASGRP1* overexpression promotes the differentiation of C2C12 cells (A) Expression levels of differentiation marker genes during differentiation. (B) The relative expression level of RASGRP1 was increased significantly and then decreased during differentiation. (C) The relative expression levels of RASGRP1 and differentiation marker genes after RASGRP1 overexpression. (D) The relative protein expression of MyHC, MyoD and MyoG after RASGRP1 overexpression. (E) MyHC immunofluorescence assay after RASGRP1 overexpression in C2C12 cells. Scale bar: 50 μm. *P<0.05, **P <0.01.

To explore the function of *RASGRP1* in C2C12 cell differentiation, we examined the expression profiles of *RASGRP1* as well as the *MyHC* and *MyoG* genes during myogenic differentiation. The qPCR results demonstrated that the expression of the *RASGRP1* gene was significantly upregulated during the differentiation of C2C12 cells, while the expressions of the *MyHC* gene and *MyoG* gene were also upregulated significantly ( Figure 4A,B), indicating that *RASGRP1* regulated the differentiation of C2C12 cells. Then, we overexpressed *RASGRP1* to explore its regulatory effect on C2C12 cell differentiation. qPCR results demonstrated that the expression of *RASGRP1* and differentiation marker genes ( *MyoG*, *MyoD* and *MyHC*) was significantly increased after *RASGRP1* overexpression ( Figure 4C). The relative protein expressions of *MyoG* and *MyHC* were markedly increased after *RASGRP1* overexpression ( Figure 4D). The MyHC immunofluorescence assay results showed that 22.23% more fusion myotubes were present in the cells overexpressing *RASGRP1* than in the control group ( Figure 4E). These results suggested that *RASGRP1* enhanced the differentiation of C2C12 cells.

**Figure 5:**
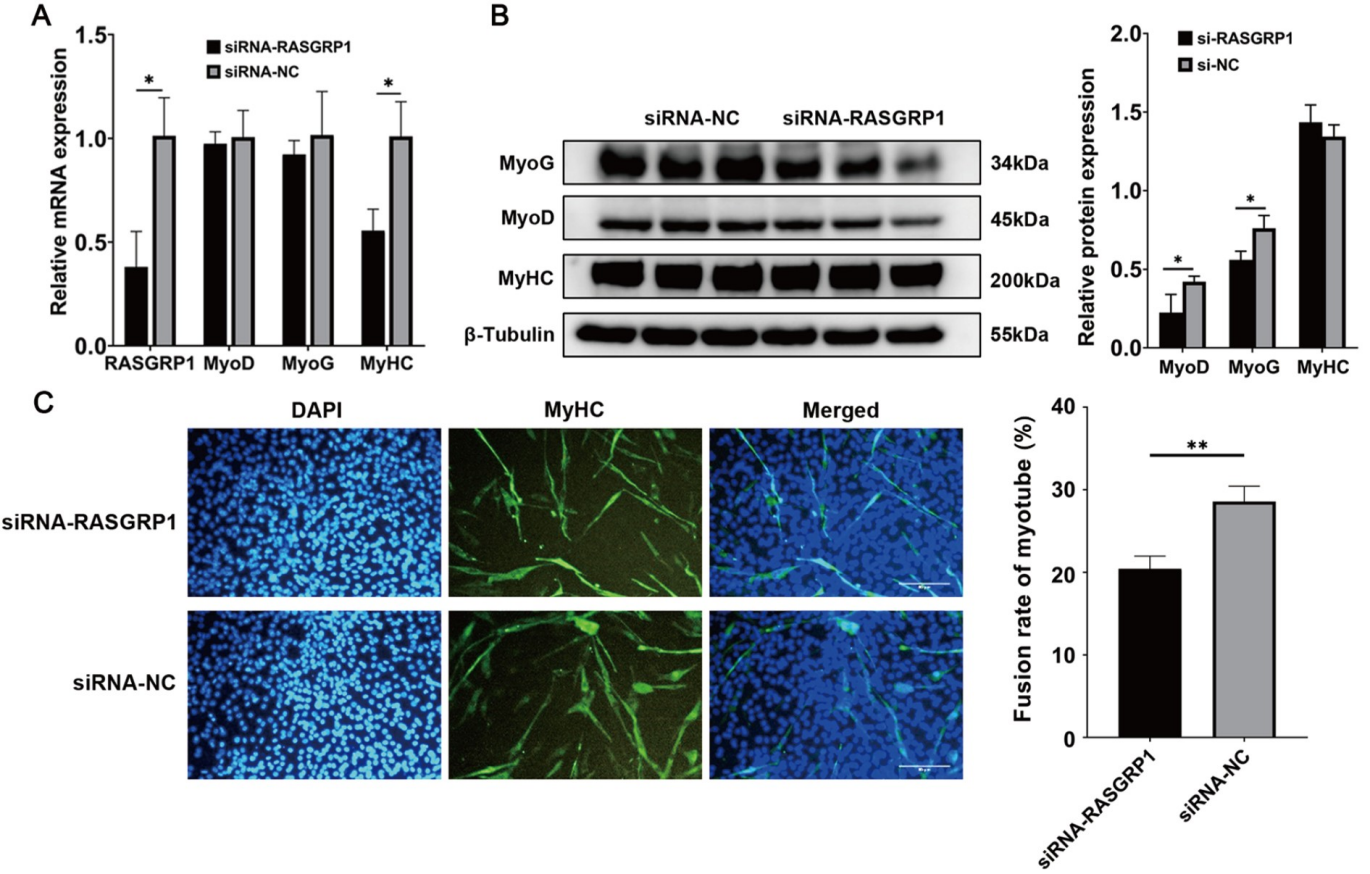
Figure 5 *RASGRP1* knockdown inhibits the differentiation of C2C12 cells (A) The relative expressions of RASGRP1 and differentiation marker genes after RASGRP1 knockdown. (B) The relative protein expression levels of MyHC, MyoD and MyoG after RASGRP1 knockdown. (C) MyHC immunofluorescence assay after RASGRP1 knockdown in C2C12 cells. Scale bar: 50 μm. *P<0.05, **P<0.01.

To further verify the effect of *RASGRP1* in regulating skeletal cell differentiation, we performed *RASGRP1* knockdown experiments by siRNA in C2C12 cells. After transfection with siRNA for three days, total RNA and protein were extracted. The qPCR results demonstrated that the relative expressions of the *RASGRP1* and *MyHC* genes were significantly decreased after siRNA transfection ( Figure 5A). The relative protein expressions of the *MyoD* and *MyoG* genes were also decreased after *RASGRP1* knockdown ( Figure 5B). The MyHC immunofluorescence assay results showed that there were 8.14% fewer fusion myotubes in the cells with *RASGRP1* gene knockdown than in the control group ( Figure 5C). All these results indicated that *RASGRP1* knockdown inhibited C2C12 cell differentiation.

### *RASGRP1* promoted the proliferation and differentiation of PSCs

**Figure 6:**
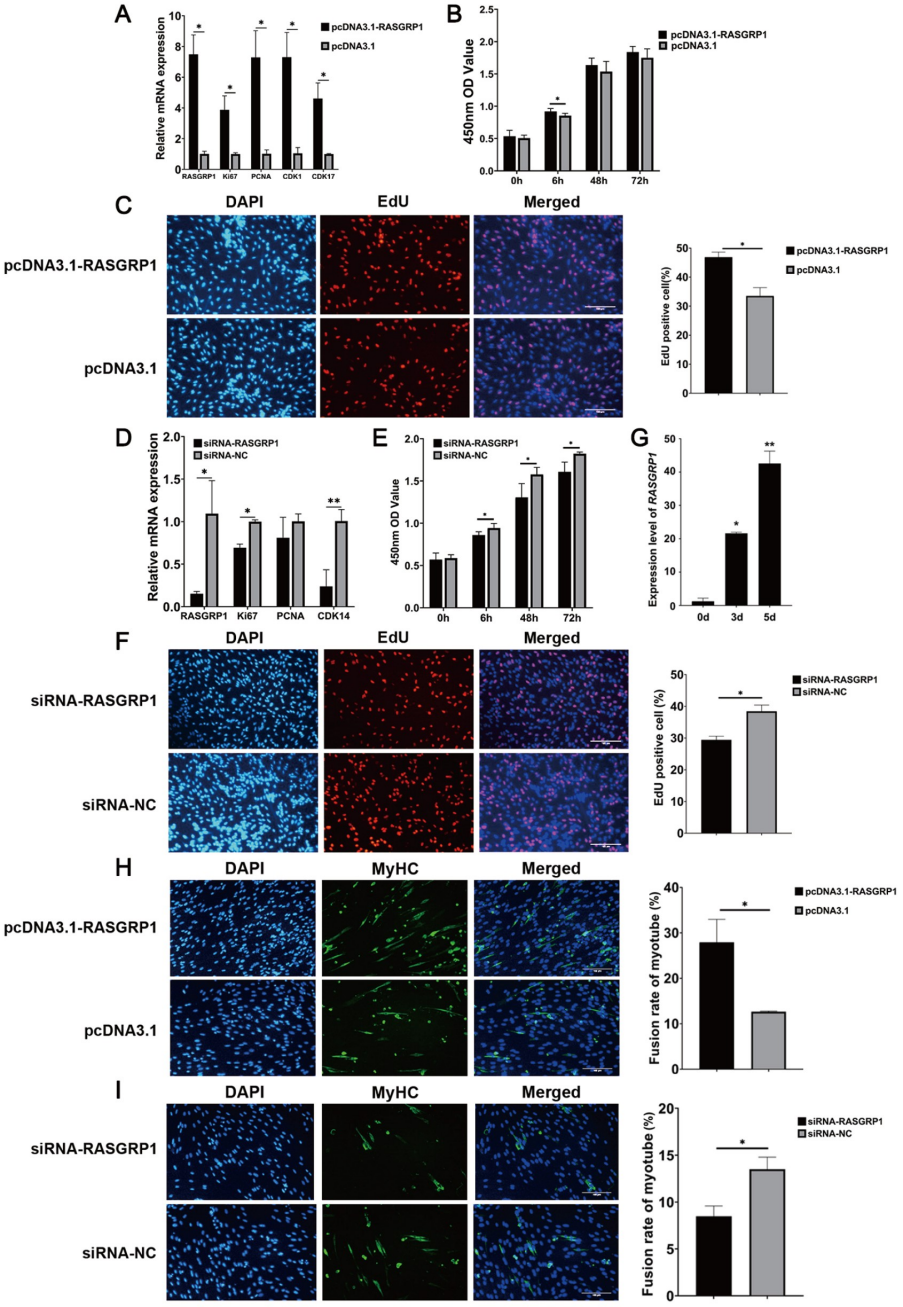
Figure 6 *RASGRP1* promotes the proliferation and differentiation of PSCs (A) The relative expression levels of RASGRP1 and proliferation marker genes after RASGRP1 overexpression. (B) Detection of PSC viability by CCK-8 assay at 6 h, 48 h and 72 h after RASGRP1 overexpression. (C) EdU staining after RASGRP1 overexpression in PSCs. Scale bar: 100 μm. (D) The relative expression levels of RASGRP1 and proliferation marker genes after RASGRP1 knockdown. (E) Detection of PSC viability at 6 h, 48 h and 72 h after RASGRP1 knockdown. (F) EdU staining after RASGRP1 knockdown in PSCs. Scale bar: 100 μm. (G) The expression of RASGRP1 at different differentiation stages of PSCs. (H) MyHC immunofluorescence assay after RASGRP1 overexpression in PSCs. Scale bar: 100 μm. (I) MyHC immunofluorescence assay after RASGRP1 knockdown in PSCs. Scale bar: 100 μm. *P<0.05, **P<0.01.

To investigate the role of *RASGRP1* in PSC proliferation and differentiation, we also studied the function of *RASGRP1* in pigs. After *RASGRP1* overexpression, the qPCR results demonstrated that the relative expressions of the *RASGRP1* gene and proliferation marker genes were significantly increased in PSCs ( Figure 6A). CCK-8 assay demonstrated that *RASGRP1* overexpression also significantly promoted the proliferation of PSCs ( Figure 6B). EdU staining showed that *RASGRP1* overexpression dramatically increased the percentage of EdU ^+^ cells ( Figure 6C). Knockdown of *RASGRP1* led to a significant decrease in the expressions of *RASGRP1* gene and proliferation marker genes ( Figure 6D). CCK-8 assay demonstrated that *RASGRP1* knockdown significantly reduced the proliferative activity of PSCs ( Figure 6E). EdU staining showed that *RASGRP1* knockdown significantly reduced the percentage of EDU ^+^ cells ( Figure 6F). We also detected the role of *RASGRP1* in PSC differentiation. In PSCs, the expression level of the *RASGRP1* gene was significantly increased during differentiation ( Figure 6G **)**. MyHC immunofluorescence assay results revealed that the fusion rate of myotubes was markedly increased/decreased after *RASGRP1* overexpression/knockdown ( Figure 6H,I). These findings suggested that *RASGRP1* stimulated the proliferation and differentiation of PSCs.

## Discussion

The *RASGRP1* gene is one of the Ras gene families whose proteins can coordinate responses within each cell by detecting signals from other parts of the body [ 46– 48] and self-regulate once their activity is altered, which may lead to the occurrence of cancer and developmental diseases [49]. Increasing numbers of studies have revealed a connection between the *RASGRP1* gene and muscle growth. RASGRP1 can affect the body size trait of pigs and exhibits a strong positive correlation with the body height and tube circumference traits of Suhuai pigs [50]. In addition, deletion of the *RASGRP1* gene inhibits the activation of ERK, which is a part of the classic MAPK/ERK pathway for regulating skeletal muscle proliferation [51]. Furthermore, *RASGRP1* was reported to be related to the regulation of p-ERK in the PPAR β/δ signaling pathway [52], which is widely involved in regulating myogenic and adipogenic differentiation [53]. These results indicated that *RASGRP1* may be involved in muscle development. However, the detailed function of *RASGRP1* in myogenesis is largely unknown.

H3K27me3 modification is widely distributed throughout the genome of myoblasts or myotubes and regulates muscle development as a silent marker mainly located at the promoter of genes [ 54, 55]. Previous studies also demonstrated that myogenic transcription factors were repressed by H3K27me3 during cell proliferation in PSCs, while H3K27me3 depletion promoted myogenic differentiation [55]. Our previous sequencing results [36] revealed that *RASGRP1* has a significant H3K27me3 enrichment peak in its promoter. Thus, we aimed to investigate the function of *RASGRP1* in myogenesis and explore the regulatory function of H3K27me3 in *RASGRP1*. In this study, we confirmed that the enrichment level of H3K27me3 on the *RASGRP1* gene decreased in pig embryonic skeletal muscle at E33, E65 and E90, which showed a significant negative correlation with *RASGRP1* gene expression during pig embryonic muscle development.

We then examined the functions of the *RASGRP1* gene in myogenesis. Since cell maturation is based on myoblast proliferation and myoblast differentiation is essential for muscle development and maturity, we examined the expression changes of some proliferation markers after loss/gain of *RASGRP1* both in C2C12 cells and PSCs, such as *Ki67*, cyclin-dependent kinases (CDKs) and proliferating cell nuclear antigen (PCNA). *Ki67* is often used as a key indicator and marker gene for cell proliferation in clinical studies [56], and CDKs participate in the regulation of the cell proliferation cycle [57]. PCNA plays an important role in cell replication and promotes cell proliferation. Our results showed that *RASGRP1* significantly promoted the relative expression of the above proliferation marker genes, which indicated its positive effect on cell proliferation. As expected, consistent results were obtained in cell proliferation assays. We found that the expression of the *RASGRP1* gene increased continuously in the process of myoblast differentiation, as did that of myocyte differentiation marker genes. After overexpression of *RASGRP1*, the relative expressions of differentiation marker factors were also upregulated significantly. The results of the MyHC immunofluorescence experiment showed that the number of differentiated myotube fusions was increased, indicating the promotion of cell differentiation. Consistently, the opposite trend was observed after *RASGRP1* knockdown. These results suggested that *RASGRP1* regulates skeletal muscle development by affecting the expressions of proliferation and differentiation factors, and this function may be conserved in mice and pigs.

In conclusion, we found that the promoter of the *RASGRP1* gene is enriched by H3K27me3. The loss of H3K27me3 enrichment in *RASGRP1* can promote its transcriptional activity during the development of skeletal muscle. Meanwhile, up/downregulation of *RASGRP1* expression can promote/repress the proliferation and differentiation of PSCs and C2C12 cells. These results suggest that *RASGRP1* has a positive regulatory role in myogenesis.

## Supporting information

ABBS-2023-425_XML-online

425FigS1-TabS1

## Supplementary Data

Supplementary data is available at *Acta Biochimica et Biphysica Sinica* online.
