# Supplementary material for: RASGRP1 targeted by H3K27me3 regulates myoblast proliferation and differentiation in mice and pigs : RASGRP1 regulates myoblast proliferation and differentiation
Source: Acta Biochim Biophys Sin (Shanghai). 2024 Feb 28;56(3):452–61. doi: 10.3724/abbs.2024011 (PMC10984873; doi:10.3724/abbs.2024011)
Supplement: ABBS-2023-425_XML-online [file ABBS-2023-425_XML-online.pdf]

Acta Biochim Biophys Sin 2024, 56(3): 1–10
https://doi.org/10.3724/abbs.2024011
Advance Access Publication Date: 28 February 2024
Original Article

Original Article

RASGRP1 targeted by H3K27me3 regulates myoblast proliferation and differentiation in mice and pigs

Liyao Xiao[1,†], Jiaxin Qiao[1,†], Yiyang Huang[1], Baohua Tan[1], Linjun Hong[1], Zicong Li[1,2,3,4], Gengyuan Cai[1,5], Zhenfang Wu[1,2,3,4,5], Enqin Zheng[1], Shanshan Wang[1,6,*], and Ting Gu[1,*]

[1]National Engineering Research Center for Breeding Swine Industry, College of Animal Science, South China Agricultural University, Guangzhou 510000, China, [2]State Key Laboratory for Conservation and Utilization of Subtropical Agro-bioresources, Guangzhou 510000, China, [3] Guangdong Provincial Laboratory of Lingnan Modern Agricultural Science and Technology, Guangzhou 510000, China, [4]Guangdong Provincial Key Laboratory of Agro-animal Genomics and Molecular Breeding, Guangzhou 510000, China, [5]Guangdong Wens Breeding Swine Technology Co., Ltd., Yunfu 527400, China, and [6]College of Life Science, Hubei University, Wuhan 430000, China

[†]These authors contributed equally to this work.

*Correspondence address. Tel: +86-20-85281547; E-mail: ws15907163745@163.com (S.W.) / E-mail: tinggu@scau.edu.cn (T.G.)

Received 9 September 2023 Accepted 14 November 2023

Abstract

Skeletal muscle is not only the largest organ in the body that is responsible for locomotion and exercise but also crucial for maintaining the body's energy metabolism and endocrine secretion. The trimethylation of histone H3 lysine 27 (H3K27me3) is one of the most important histone modifications that participates in muscle development regulation by repressing the transcription of genes. Previous studies indicate that the *RASGRP1* gene is regulated by H3K27me3 in embryonic muscle development in pigs, but its function and regulatory role in myogenesis are still unclear. In this study, we verify the crucial role of H3K27me3 in *RASGRP1* regulation. The gain/loss function of *RASGRP1* in myogenesis regulation is performed using mouse myoblast C2C12 cells and primarily isolated porcine skeletal muscle satellite cells (PSCs). The results of qPCR, western blot analysis, EdU staining, CCK-8 assay and immunofluorescence staining show that overexpression of *RASGRP1* promotes cell proliferation and differentiation in both skeletal muscle cell models, while knockdown of *RASGRP1* leads to the opposite results. These findings indicate that *RASGRP1* plays an important regulatory role in myogenesis in both mice and pigs.

Key words RASGRP1, H3K27me3, cell proliferation, myoblasts, skeletal muscle

Introduction

Skeletal muscle is not only the largest organ responsible for animal locomotion and exercise capacity but also crucial for maintaining the body's energy metabolism and endocrine secretion [1–3]. In livestock-producing fields, skeletal muscle is of particular importance, as meat is the main animal product, in addition to animal fur, milk and others. Increasing evidence indicates that epigenetic regulators participate in the development of skeletal muscle.

Epigenetic regulators such as DNA methylation, histone methylation, microRNAs (miRNAs) and long noncoding RNAs (lncRNAs) play vital roles in regulating myogenesis by affecting the transcription process or posttranscriptional modifications of RNAs [4–7]. Among them, the trimethylation of histone H3 lysine 27 (H3K27me3) is a well-known repressive marker involved in various biological processes [8–10]. H3K27me3 participates in the regulation of myogenic differentiation by silencing muscle-specific genes and cell cycle genes [11–14]. The loss of H3K27me3 in the gene body, especially in the promoter of genes, repressed the differentiation of skeletal muscle cells [15–17]. The methyltransferase of H3K27me3, named enhancer of zeste homolog 2 (EZH2), which is a subunit of polycomb repressive complex 2 (PRC2), can also directly or indirectly regulate the expressions of myogenic genes [18,19]. During myogenic differentiation, the phosphorylated EZH2 enhancer induces a shift from H3K4me3 to H3K27me3 on the *Pax7* promoter to downregulate gene expression [20]. In contrast, the decrease in H3K27me3 is related to genes that are more actively transcribed [21].

The *RASGRP1* gene is a member of the Ras gene family, which is highly expressed in T cells [22]. It has been reported that dysregulation of *RASGRP1* leads to the occurrence of cancers

© The Author(s) 2023. This is an open access article distributed under the terms of the Creative Commons Attribution License (https://creativecommons.org/licenses/by/4.0/).

[23–28] and other diseases [29–33] related to the body's immunity [34,35]. Our previous study found that *RASGRP1* may regulate pig embryonic myogenesis, which was regulated by H3K27me3 through chromatin immunoprecipitation-sequencing (ChIP-seq) and RNA-sequencing of the longissimus dorsi muscle in Duroc pig embryos at gestation days 33 (E33), 65 (E65), and 90 (E90) [36]. However, the detailed function of this gene in regulating myogenesis is still unclear.

In this study, we first confirmed that the expression of the *RASGRP1* gene was regulated by H3K27me3 enrichment in its promoter during skeletal muscle development. Then, we studied the function of *RASGRP1* during cell proliferation and differentiation in both mouse C2C12 myoblasts and PSCs. Our results demonstrated that *RASGRP1* is regulated by H3K27me3 and promotes myogenesis in pigs and mice.

Materials and Methods

Animals

Pigs, including Duroc sows for embryonic skeletal muscle collection and newborn piglets for isolating porcine skeletal muscle satellite cells, were purchased from Guangdong Wen's Foodstuffs Group (Yunfu, China). All animal experiments were conducted following the requirements for the Care and Use of Laboratory Animals by the Ethics Committee of the Laboratory Animal Center of South China Agricultural University, Guangzhou, China (Permit Number 2021F036, Permit Date 2 March 2021) [37].

Cell culture

C2C12 cells were purchased from the Cell Bank of the Chinese Academy of Sciences (Shanghai, China). Dulbecco's modified Eagle's medium (DMEM) high glucose (Gibco, Grand Island, USA) and 10% fetal bovine serum (FBS; Gibco) was used for cell culture. For cell differentiation, DMEM high glucose with 2% horse serum (Gibco) was used. The cells were cultured with 5% CO_2 at 37°C.

Isolation and culture of porcine skeletal muscle satellite cells

Porcine skeletal muscle satellite cells (PSCs) were isolated from one-week-old piglets and cultivated according to protocols described in previous studies [38,39]. The tissues were digested with 2 mg/mL type II collagenase (Sigma-Aldrich, Darmstadt, Germany) and then placed in a 37°C incubator for 2.5 h. Then, the digestion was stopped with an equal volume of RPMI 1640 medium (Gibco) containing 1% penicillin-streptomycin (P-S; Gibco). Then, 100, 200, and 400-mesh sieves were used to filter the cell suspension. Cell proliferation medium was prepared according to a previous study and used to resuspend cells [39]. Differential adhesion was used to obtain purified cells after 2 h of culture. When cells reached 70%–80% confluence, differentiation medium consisting of DMEM high glucose with 2% horse serum and 1% P-S was used to induce cell differentiation.

Plasmid construction, small interfering RNA synthesis, and transfection

The coding sequence of the porcine *RASGRP1* gene was cloned *in vitro* by PCR amplification and connected to the linear pcDNA3.1 (+) vector (Sangon, Shanghai, China) to produce the pcDNA3.1-*RASGRP1* plasmid. The primers for the *RASGRP1* CD sequence were as follows: forward primer 5′-TACCGAGCTCGGATCCATGGGCACCCTGGGCAAG-3′ and reverse primer 5′-GATATCTGCAGAATTCCTAAGAACAGTCACCGTGCTCCATC-3′. Small interfering RNAs (siRNAs) against pig *RASGRP1* and mouse *RASGRP1* genes were designed and synthesized by GenePharma (Shanghai, China). The siRNA sequences were as follows: Sus-siRNA-*RASGRP1* (sense 5′-CCCAGUGGGUUCAACUCAUTT, antisense 5′-AUGAGUUGAACCCACUGGGTT-3′), Mus-siRNA-*RASGRP1* (sense 5′-GGACCUCAUAUCCCUGUAUTT-3′, antisense 5′-AUACAGGGAUAUGAGGUCCTT-3′), and siRNA negative control (sense: 5′-UUCUCCGAACGUGUCACGUTT-3′, antisense: 5′-ACGUGACACGUUCGGAGAATT-3′). Lipofectamine 3000 (Invitrogen, Carlsbad, USA) was used to conduct cell transfection following the manufacturer's protocol.

Quantitative real-time polymerase chain reaction (qPCR)

Total RNA kit ll (Omega Biotek, Norcross, USA) was used to harvest the total RNA following the manufacturer's protocol. cDNA was prepared using a PrimeScript RT reagent kit (TaKaRa, Tokyo, Japan) and used to perform qPCR in a Quant Studio 7 Flex system (Thermo Fisher, Scientific, Waltham, USA). All primers used are presented in Supplementary Table S1.

Chromatin immunoprecipitation (ChIP)

ChIP was conducted according to the protocol in a previous study [40]. Briefly, micrococcal nuclease was used to fragment chromatin. After that, the chromatin fragments were incubated with magnetic beads (Biogle, Wuxi, China). The DNA-bead complex was immunoprecipitated with anti-H3K27me3 antibody (Millipore, Billerica, USA) or negative control IgG. The primers for the promoter of the *RASGRP1* gene used for ChIP-qPCR were as follows: forward primer 5′-CTCTCCGAATTCCCCATTGTG-3′ and reverse primer 5′-AAATCAGAGCTGCATCCAC-3′.

Western blot analysis

The concentration of protein harvested from cells by RIPA buffer with 1% PMSF (Beyotime, Shanghai, China) was determined using a BCA Assay Kit (Thermo Fisher Scientific). Western blot analysis was performed following a previous study [39]. The antibodies used were as follows: Ki67 (ab16667; 1:1000; Abcam, Cambridge, UK), CDK2 (PA1547; 1:1000; Boster, Pleasanton, USA), MyoG (sc-12732; 1:500; Santa Cruz Biotechnology, Santa Cruz, USA), MyoD (sc-377460; 1:500; Santa Cruz Biotechnology), MyHC (sc-376157; 1:1000; Santa Cruz Biotechnology), β-Tubulin (GB11017; 1:1000; Servicebio, Wuhan, China), goat anti-mouse IgG (A0216; 1:3000; Beyotime) and goat anti-rabbit IgG (A0208; 1:3000; Beyotime).

Cell proliferation and cell cycle assays

For flow cytometry analysis, C2C12 cells stored at –20°C were fixed in 70% (v/v) ethanol overnight and treated at 4°C in 50 mg/mL propidium iodide for 30 min. Cell cycle detection was conducted in accordance with a previous study [39] by using a flow cytometer (Becton Dickinson, Franklin Lakes, USA). The cells were transfected with *RASGRP1* siRNA or overexpression vectors when they reached approximately 50% confluence. CCK-8 assays (Yeasen, Shanghai, China) were conducted following the manufacturer's protocol after transfection. EdU staining was performed following the manufacturer's protocol (RiboBio, Guangzhou, China). Cells were to fixed and permeabilized separately with 4% paraformaldehyde and 0.5% Triton X-100, respectively. DAPI was used to stain

cell nuclei. All images were captured with the TE2000U fluorescence microscope imaging system (Nikon, Tokyo, Japan).

Immunofluorescence staining

Cells seeded in a 24-well plate were transfected with *RASGRP1* siRNA or overexpression vectors when they reached approximately 50%–70% confluence. Then, 4% paraformaldehyde and 0.5% Triton X-100 were separately used to fix and permeabilize the cells for 30 min three days after transfection. Then, the cells were blocked in QuickBlock Buffer (Beyotime) at 37°C for 2 h and treated with the anti-MyHC antibody (sc-376157; 1:200; Santa Cruz Biotechnology) at 4°C overnight. Then, the cells were incubated with Alexa Fluor 488 fluorescent antibody (1:500; Thermo Fisher Scientific) after wash with PBS (Servicebio). DAPI was used to stain cell nuclei. All images were captured with the TE2000U fluorescence microscope imaging system.

Statistical analysis

Data are presented as the mean ± standard error of the mean (SEM). The comparative threshold cycle ($2^{-\Delta\Delta ct}$) method was used to quantify the relative mRNA levels [38,41]. Statistical analysis between different groups was performed by using two-tailed Student's *t*-test or one-way analysis of variance (ANOVA) in SPSS software (version 22.0). ImageJ software was used for the quantification of positively-stained cells and the visualization of western blots. All experiments were performed in triplicate. $P < 0.05$ indicated significant difference.

Results

RASGRP1 is targeted by H3k27me3

We collected longissimus dorsi muscles on gestation days 33, 65, and 90, which represent three critical developmental time points for embryonic myogenesis [42–45]. ChIP-qPCR assay in skeletal muscles at E33, E65, and E90 showed that the enrichment of H3K27me3 in the *RASGRP1* promoter was highest at E33 and lowest at E90 (Figure 1A). The mRNA expression of the *RASGRP1* gene significantly increased during embryonic development (Figure 1B and Supplementary Figure S1), while its expression was pretty low in adult skeletal muscle. These results indicated that during embryonic porcine myogenesis, increased *RASGRP1* expression might be specifically regulated by H3K27me3.

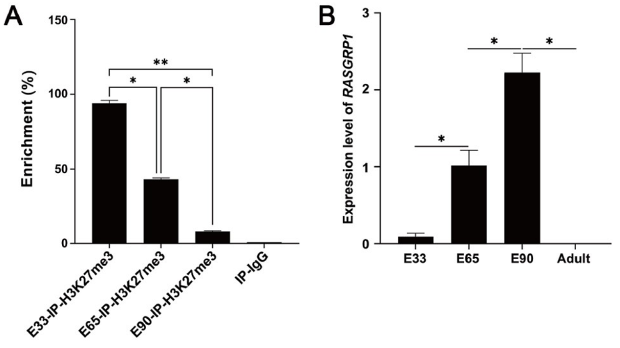

Figure 1. *RASGRP1* is targeted by H3K27me3 and upregulated in the longissimus dorsi muscle of Duroc pig embryos at gestation days 33 (E33), 65 (E65), and 90 (E90) (A) The enrichment of H3K27me3 in the *RASGRP1* promoter at E33, E65, and E90. (B) The expression level of *RASGRP1* in pig skeletal muscle at E33, E65, E90 and the adult period. β-Actin was used as a control. $*P < 0.05$, $**P < 0.01$.

RASGRP1 promotes the proliferation of C2C12 cells

To investigate the role of the *RASGRP1* gene in C2C12 cell proliferation, we performed gain- and loss-of-function analyses of the *RASGRP1* gene. We overexpressed the *RASGRP1* gene in C2C12 cells, and the qPCR results demonstrated that the relative expression of the *RASGRP1* gene and the proliferation marker genes *CDK2* and *CDK14* were significantly increased (Figure 2A). CCK-8 assay demonstrated that *RASGRP1* overexpression also significantly promoted the proliferation of C2C12 cells (Figure 2B). The relative protein expressions of the proliferation marker genes *Ki67* and *CDK2* were also increased, according to western blot analysis (Figure 2C). EdU staining revealed that *RASGRP1* overexpression dramatically increased the percentage of EdU-positive (EdU^+) cells (Figure 2D). In addition, flow cytometry analysis showed that there were 5.33% more S-phase cells among the cells overexpressing *RASGRP1* than in the pcDNA3.1(+) control group (Figure 2E). All the above results indicated that *RASGRP1* promoted C2C12 cell proliferation.

To verify the effect of *RASGRP1* overexpression on C2C12 cell proliferation, we then knocked down *RASGRP1* by siRNA in C2C12 cells and detected its effect on C2C12 cell proliferation. The qPCR results showed that the relative expression of *RASGRP1* as well as *CDK2* and *CDK14* was significantly reduced after *RASGRP1* knockdown (Figure 3A). CCK-8 assay demonstrated that *RASGRP1* knockdown also dramatically reduced the proliferation activity of C2C12 cells (Figure 3B). The relative protein expression of the *Ki67* gene was markedly decreased (Figure 3C). EdU staining revealed that *RASGRP1* knockdown dramatically reduced the percentage of EdU^+ cells (Figure 3D). These results indicated that *RASGRP1* knockdown inhibited the proliferation of C2C12 cells, which was opposite to the positive effect of *RASGRP1* overexpression on the proliferation of C2C12 cells.

RASGRP1 promotes the differentiation of C2C12 cells

To explore the function of *RASGRP1* in C2C12 cell differentiation, we examined the expression profiles of *RASGRP1* as well as the *MyHC* and *MyoG* genes during myogenic differentiation. The qPCR results demonstrated that the expression of the *RASGRP1* gene was significantly upregulated during the differentiation of C2C12 cells, while the expressions of the *MyHC* gene and *MyoG* gene were also upregulated significantly (Figure 4A,B), indicating that *RASGRP1* regulated the differentiation of C2C12 cells. Then, we overexpressed *RASGRP1* to explore its regulatory effect on C2C12 cell differentiation. qPCR results demonstrated that the expression of *RASGRP1* and differentiation marker genes (*MyoG*, *MyoD* and *MyHC*) was significantly increased after *RASGRP1* overexpression (Figure 4C). The relative protein expressions of *MyoG* and *MyHC* were markedly increased after *RASGRP1* overexpression (Figure 4D). The MyHC immunofluorescence assay results showed that 22.23% more fusion myotubes were present in the cells overexpressing *RASGRP1* than in the control group (Figure 4E). These results suggested that *RASGRP1* enhanced the differentiation of C2C12 cells.

To further verify the effect of *RASGRP1* in regulating skeletal cell differentiation, we performed *RASGRP1* knockdown experiments by siRNA in C2C12 cells. After transfection with siRNA for three days, total RNA and protein were extracted. The qPCR results demonstrated that the relative expressions of the *RASGRP1* and *MyHC* genes were significantly decreased after siRNA transfection

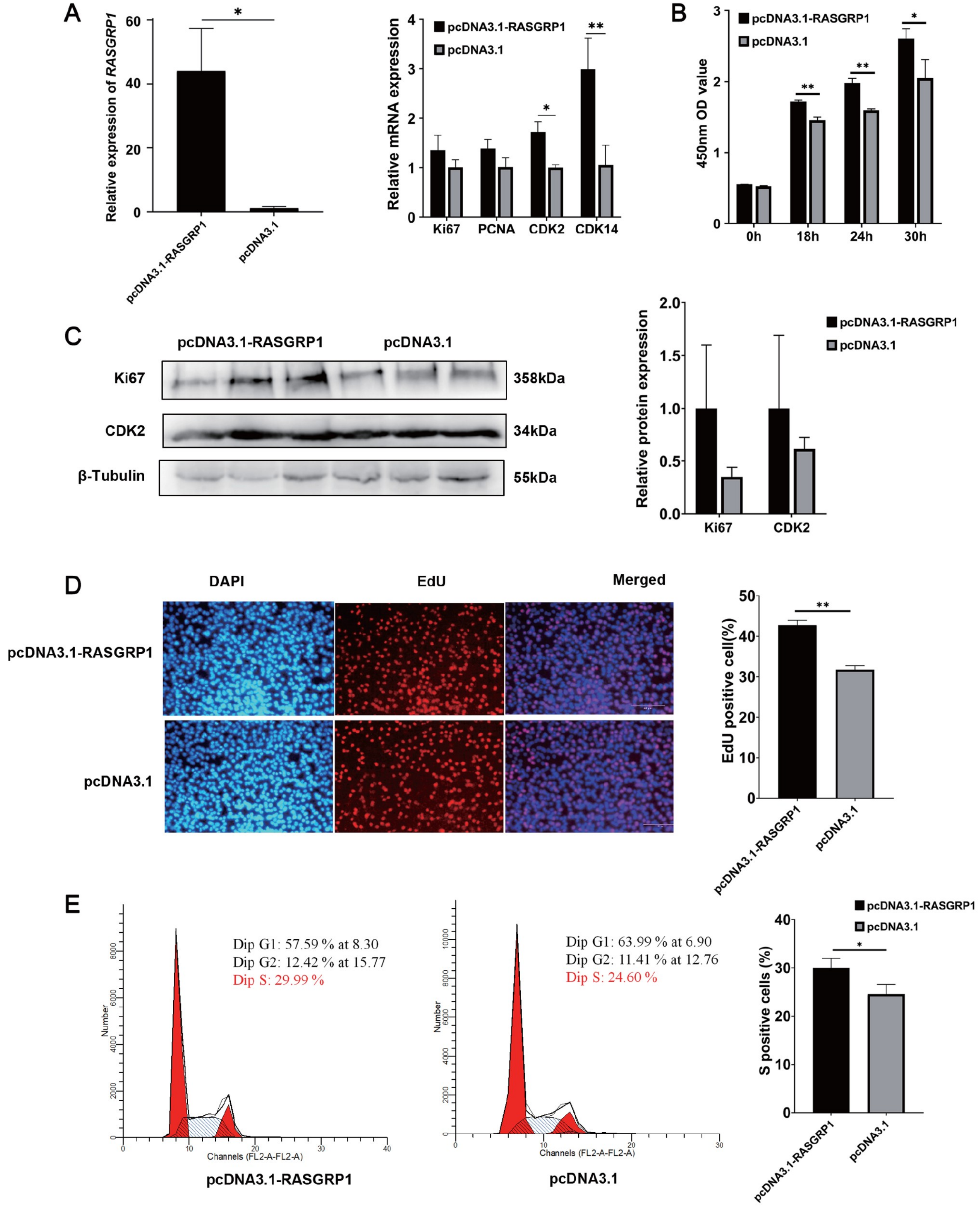

Figure 2. *RASGRP1* overexpression promotes the proliferation of C2C12 cells (A) The relative expression levels of *RASGRP1* and proliferation marker genes after *RASGRP1* overexpression. (B) Detection of C2C12 cell viability by CCK-8 assay at 18 h, 24 h and 30 h after *RASGRP1* overexpression. (C) The relative protein levels of the Ki67 and CDK2 genes after *RASGRP1* overexpression. (D) EdU staining after *RASGRP1* overexpression in C2C12 cells. Scale bar: 100 μm. (E) Detection of the number of S-phase cells by flow cytometry after *RASGRP1* overexpression. $^*P<0.05$, $^{**}P<0.01$.

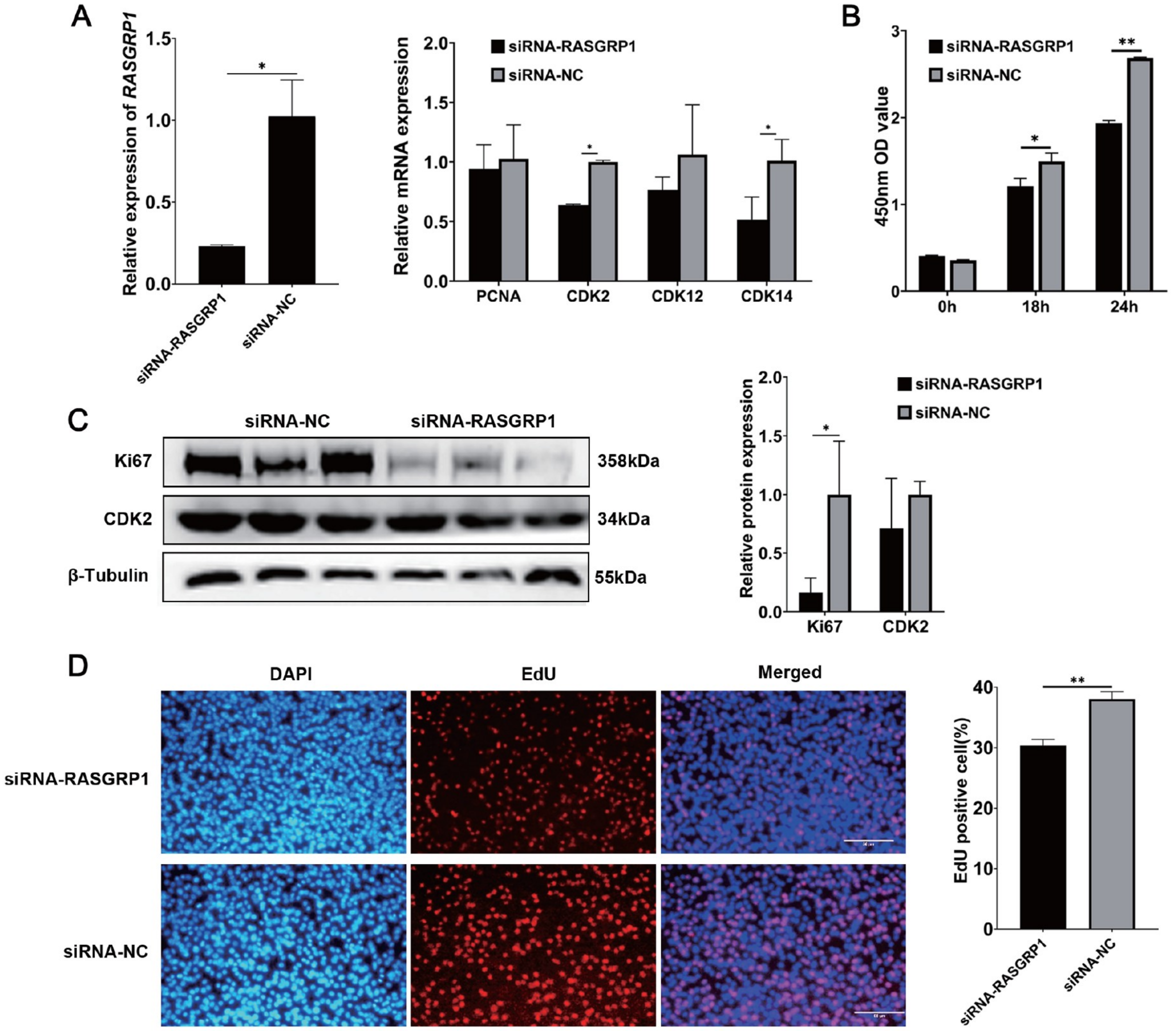

Figure 3. *RASGRP1* knockdown inhibits the proliferation of C2C12 cells (A) The relative expression levels of the RASGRP1 gene and proliferation marker genes after *RASGRP1* knockdown. (B) Detection of C2C12 cell viability by CCK-8 assay at 18 h and 24 h after *RASGRP1* knockdown. (C) The relative protein expression levels of the Ki67 and CDK2 genes after *RASGRP1* knockdown. (D) EdU staining after *RASGRP1* knockdown in C2C12 cells. Scale bar: 100 μm. *$P<0.05$, **$P<0.01$.

(Figure 5A). The relative protein expressions of the *MyoD* and *MyoG* genes were also decreased after *RASGRP1* knockdown (Figure 5B). The MyHC immunofluorescence assay results showed that there were 8.14% fewer fusion myotubes in the cells with *RASGRP1* gene knockdown than in the control group (Figure 5C). All these results indicated that *RASGRP1* knockdown inhibited C2C12 cell differentiation.

RASGRP1 promoted the proliferation and differentiation of PSCs

To investigate the role of *RASGRP1* in PSC proliferation and differentiation, we also studied the function of *RASGRP1* in pigs. After *RASGRP1* overexpression, the qPCR results demonstrated that the relative expressions of the *RASGRP1* gene and proliferation marker genes were significantly increased in PSCs (Figure 6A). CCK-8 assay demonstrated that *RASGRP1* overexpression also significantly promoted the proliferation of PSCs (Figure 6B). EdU staining showed that *RASGRP1* overexpression dramatically increased the percentage of EdU^+ cells (Figure 6C). Knockdown of *RASGRP1* led to a significant decrease in the expressions of *RASGRP1* gene and proliferation marker genes (Figure 6D). CCK-8 assay demonstrated that *RASGRP1* knockdown significantly reduced the proliferative activity of PSCs (Figure 6E). EdU staining showed that *RASGRP1* knockdown significantly reduced the percentage of EDU^+ cells (Figure 6F). We also detected the role of *RASGRP1* in PSC differentiation. In PSCs, the expression level of the *RASGRP1* gene was significantly increased during differentiation (Figure 6G). MyHC immunofluorescence assay results revealed that the fusion rate of myotubes was markedly increased/decreased after *RASGRP1* overexpression/knockdown (Figure 6H,I). These findings suggested that *RASGRP1* stimulated the proliferation and differentiation of PSCs.

Discussion

The *RASGRP1* gene is one of the Ras gene families whose proteins can coordinate responses within each cell by detecting signals from other parts of the body [46–48] and self-regulate once their activity is altered, which may lead to the occurrence of cancer and

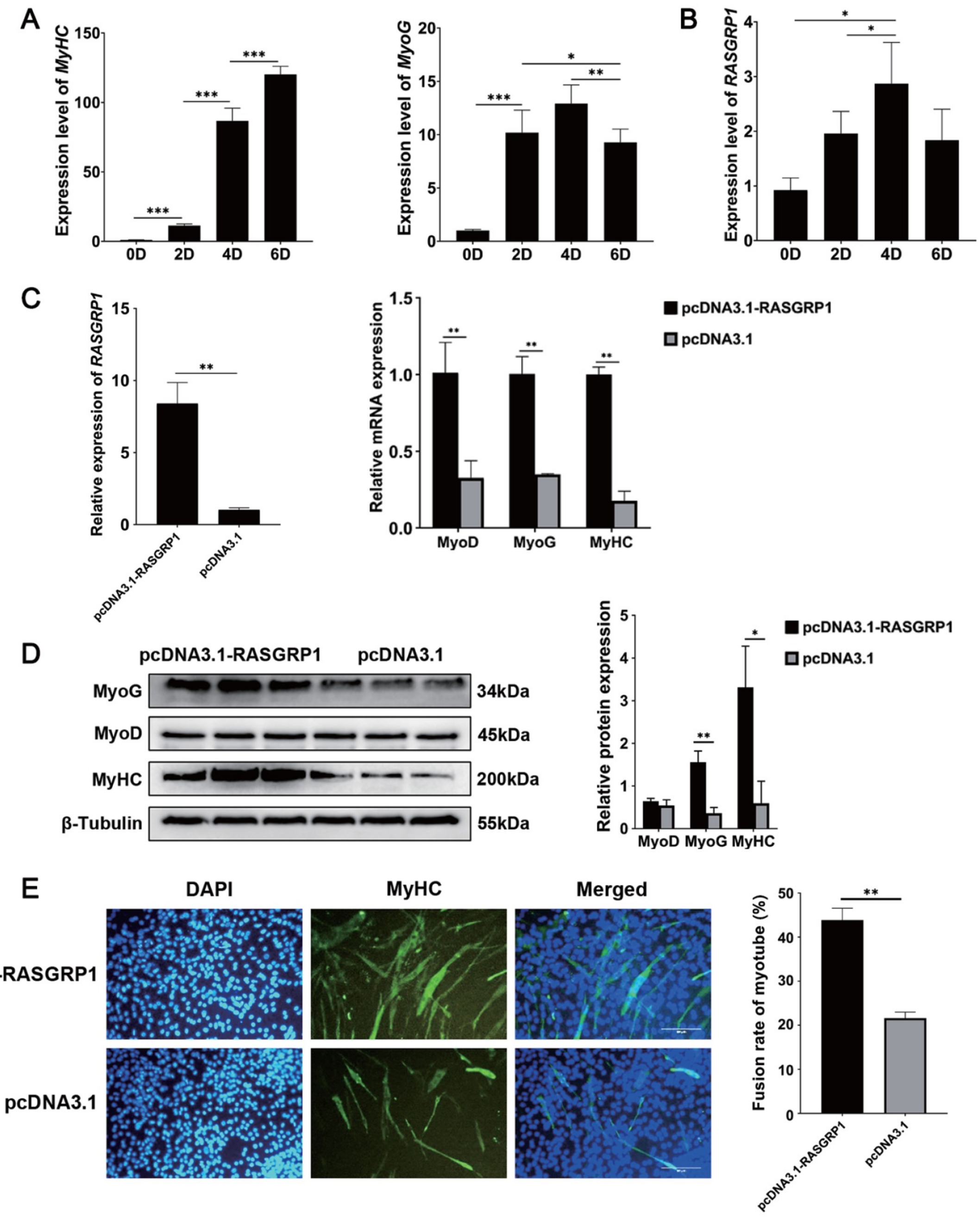

Figure 4. *RASGRP1* overexpression promotes the differentiation of C2C12 cells (A) Expression levels of differentiation marker genes during differentiation. (B) The relative expression level of *RASGRP1* was increased significantly and then decreased during differentiation. (C) The relative expression levels of *RASGRP1* and differentiation marker genes after *RASGRP1* overexpression. (D) The relative protein expression of *MyHC*, *MyoD* and *MyoG* after *RASGRP1* overexpression. (E) MyHC immunofluorescence assay after *RASGRP1* overexpression in C2C12 cells. Scale bar: 50 μm. **P* < 0.05, ***P* < 0.01.

developmental diseases [49]. Increasing numbers of studies have revealed a connection between the *RASGRP1* gene and muscle growth. RASGRP1 can affect the body size trait of pigs and exhibits a strong positive correlation with the body height and tube circumference traits of Suhuai pigs [50]. In addition, deletion of the *RASGRP1* gene inhibits the activation of ERK, which is a part of the classic MAPK/ERK pathway for regulating skeletal muscle proliferation [51]. Furthermore, *RASGRP1* was reported to be related to the regulation of p-ERK in the PPAR β/δ signaling pathway [52], which is widely involved in regulating myogenic and adipogenic differentiation [53]. These results indicated that *RASGRP1* may be involved in muscle development. However, the detailed function of *RASGRP1* in myogenesis is largely unknown.

H3K27me3 modification is widely distributed throughout the genome of myoblasts or myotubes and regulates muscle development as a silent marker mainly located at the promoter of genes [54,55]. Previous studies also demonstrated that myogenic transcription factors were repressed by H3K27me3 during cell prolifera-

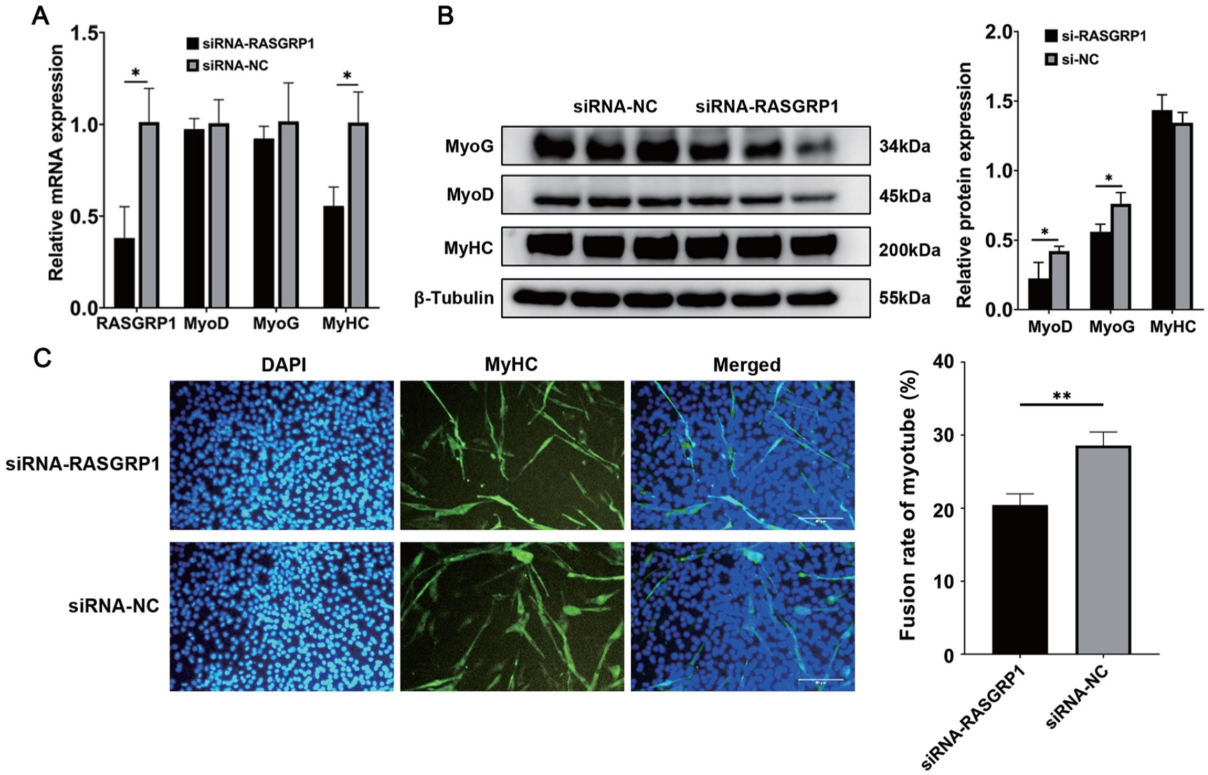

Figure 5. *RASGRP1* knockdown inhibits the differentiation of C2C12 cells (A) The relative expressions of *RASGRP1* and differentiation marker genes after *RASGRP1* knockdown. (B) The relative protein expression levels of *MyHC*, *MyoD* and *MyoG* after *RASGRP1* knockdown. (C) MyHC immunofluorescence assay after *RASGRP1* knockdown in C2C12 cells. Scale bar: 50 μm. $^{*}P<0.05$, $^{**}P<0.01$.

tion in PSCs, while H3K27me3 depletion promoted myogenic differentiation [55]. Our previous sequencing results [36] revealed that *RASGRP1* has a significant H3K27me3 enrichment peak in its promoter. Thus, we aimed to investigate the function of *RASGRP1* in myogenesis and explore the regulatory function of H3K27me3 in *RASGRP1*. In this study, we confirmed that the enrichment level of H3K27me3 on the *RASGRP1* gene decreased in pig embryonic skeletal muscle at E33, E65 and E90, which showed a significant negative correlation with *RASGRP1* gene expression during pig embryonic muscle development.

We then examined the functions of the *RASGRP1* gene in myogenesis. Since cell maturation is based on myoblast proliferation and myoblast differentiation is essential for muscle development and maturity, we examined the expression changes of some proliferation markers after loss/gain of *RASGRP1* both in C2C12 cells and PSCs, such as *Ki67*, cyclin-dependent kinases (CDKs) and proliferating cell nuclear antigen (PCNA). *Ki67* is often used as a key indicator and marker gene for cell proliferation in clinical studies [56], and CDKs participate in the regulation of the cell proliferation cycle [57]. PCNA plays an important role in cell replication and promotes cell proliferation. Our results showed that *RASGRP1* significantly promoted the relative expression of the above proliferation marker genes, which indicated its positive effect on cell proliferation. As expected, consistent results were obtained in cell proliferation assays. We found that the expression of the *RASGRP1* gene increased continuously in the process of myoblast differentiation, as did that of myocyte differentiation marker genes. After overexpression of *RASGRP1*, the relative expressions of differentiation marker factors were also upregulated significantly. The results of the MyHC immunofluorescence experiment showed that the number of differentiated myotube fusions was increased, indicating the promotion of cell differentiation. Consistently, the opposite trend was observed after *RASGRP1* knockdown. These results suggested that *RASGRP1* regulates skeletal muscle development by affecting the expressions of proliferation and differentiation factors, and this function may be conserved in mice and pigs.

In conclusion, we found that the promoter of the *RASGRP1* gene is enriched by H3K27me3. The loss of H3K27me3 enrichment in *RASGRP1* can promote its transcriptional activity during the development of skeletal muscle. Meanwhile, up/downregulation of *RASGRP1* expression can promote/repress the proliferation and differentiation of PSCs and C2C12 cells. These results suggest that *RASGRP1* has a positive regulatory role in myogenesis.

Supplementary Data

Supplementary data is available at *Acta Biochimica et Biphysica Sinica* online.

Funding

This research was supported by the grants from the 2023 Key Areas Research and Development Programs - Modern Seed Industry of Guangdong Province, China (No. 2022B0202090002) and the Provincial Rural Revitalization Strategy Special Project on the Protection and Development and Utilization of Local Livestock and Poultry of Guangdong Province, China.

Conflict of Interest

The authors declare that they have no conflict of interest.

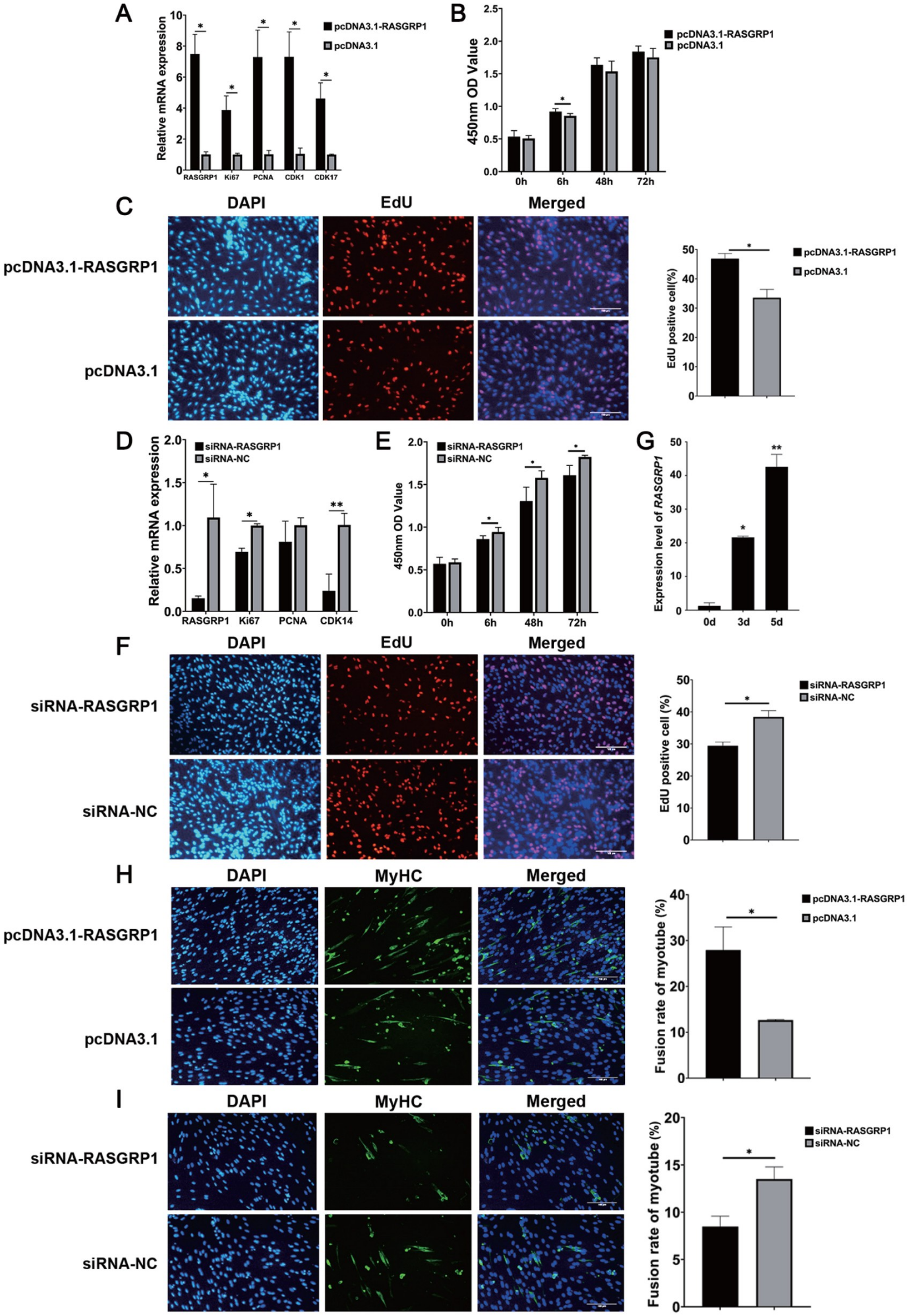

Figure 6. ***RASGRP1* promotes the proliferation and differentiation of PSCs** (A) The relative expression levels of *RASGRP1* and proliferation marker genes after *RASGRP1* overexpression. (B) Detection of PSC viability by CCK-8 assay at 6 h, 48 h and 72 h after *RASGRP1* overexpression. (C) EdU staining after *RASGRP1* overexpression in PSCs. Scale bar: 100 μm. (D) The relative expression levels of *RASGRP1* and proliferation marker genes after *RASGRP1* knockdown. (E) Detection of PSC viability at 6 h, 48 h and 72 h after *RASGRP1* knockdown. (F) EdU staining after *RASGRP1* knockdown in PSCs. Scale bar: 100 μm. (G) The expression of *RASGRP1* at different differentiation stages of PSCs. (H) MyHC immunofluorescence assay after *RASGRP1* overexpression in PSCs. Scale bar: 100 μm. (I) MyHC immunofluorescence assay after *RASGRP1* knockdown in PSCs. Scale bar: 100 μm. $^{*}P<0.05$, $^{**}P<0.01$.

References

1. Kissane RWP, Charles JP, Banks RW, Bates KT. Skeletal muscle function underpins muscle spindle abundance. *Proc R Soc B* 2022, 289: 20220622
2. Argilés JM, Campos N, Lopez-Pedrosa JM, Rueda R, Rodriguez-Mañas L. Skeletal muscle regulates metabolism via interorgan crosstalk: roles in health and disease. *J Am Med Directors Assoc* 2016, 17: 789–796
3. Pedersen BK, Febbraio MA. Muscles, exercise and obesity: skeletal muscle as a secretory organ. *Nat Rev Endocrinol* 2012, 8: 457–465
4. Li Y, Jiang J, Liu W, Wang H, Zhao L, Liu S, Li P, *et al.* microRNA-378 promotes autophagy and inhibits apoptosis in skeletal muscle. *Proc Natl Acad Sci USA* 2018, 115: E10849–E10858
5. Li Y, Zhang Y, Hu Q, Egranov SD, Xing Z, Zhang Z, Liang K, *et al.* Functional significance of gain-of-function H19 lncRNA in skeletal muscle differentiation and anti-obesity effects. *Genome Med* 2021, 13: 137
6. Jin W, Peng J, Jiang S. The epigenetic regulation of embryonic myogenesis and adult muscle regeneration by histone methylation modification. *Biochem Biophys Rep* 2016, 6: 209–219
7. Jenuwein T, Allis CD. Translating the histone code. *Science* 2001, 293: 1074–1080
8. Schuettengruber B, Martinez AM, Iovino N, Cavalli G. Trithorax group proteins: switching genes on and keeping them active. *Nat Rev Mol Cell Biol* 2011, 12: 799–814
9. Duan R, Du W, Guo W. EZH2: a novel target for cancer treatment. *J Hematol Oncol* 2020, 13: 104
10. Kimura H. Histone modifications for human epigenome analysis. *J Hum Genet* 2013, 58: 439–445
11. Asp P, Blum R, Vethantham V, Parisi F, Micsinai M, Cheng J, Bowman C, *et al.* Genome-wide remodeling of the epigenetic landscape during myogenic differentiation. *Proc Natl Acad Sci USA* 2011, 108: E149–E158
12. Blais A, van Oevelen CJC, Margueron R, Acosta-Alvear D, Dynlacht BD. Retinoblastoma tumor suppressor protein–dependent methylation of histone H3 lysine 27 is associated with irreversible cell cycle exit. *J Cell Biol* 2007, 179: 1399–1412
13. Vella S, Pomella S, Leoncini PP, Colletti M, Conti B, Marquez VE, Strillacci A, *et al.* MicroRNA-101 is repressed by EZH2 and its restoration inhibits tumorigenic features in embryonal rhabdomyosarcoma. *Clin Epigenet* 2015, 7: 82
14. Andresini O, Rossi MN, Matteini F, Petrai S, Santini T, Maione R. The long non-coding RNA Kcnq1ot1 controls maternal p57 expression in muscle cells by promoting H3K27me3 accumulation to an intragenic MyoD-binding region. *Epigenet Chromatin* 2019, 12: 8
15. Faralli H, Wang C, Nakka K, Benyoucef A, Sebastian S, Zhuang L, Chu A, *et al.* UTX demethylase activity is required for satellite cell–mediated muscle regeneration. *J Clin Invest* 2016, 126: 1555–1565
16. Seenundun S, Rampalli S, Liu QC, Aziz A, Palii C, Hong SH, Blais A, *et al.* UTX mediates demethylation of H3K27me3 at muscle-specific genes during myogenesis. *EMBO J* 2010, 29: 1401–1411
17. Li J, Zhang YS, Li N, Hu XX, Shi GQ, Liu SR, Liu N. Expression of Myogenin and MCK genes regulated by PI3K/AKT pathway. *Hereditas (Beijing)* 2013, 35: 637–642
18. Zoroddu S, Marchesi I, Bagella L. PRC2: an epigenetic multiprotein complex with a key role in the development of rhabdomyosarcoma carcinogenesis. *Clin Epigenet* 2021, 13: 156
19. Marchesi I, Giordano A, Bagella L. Roles of enhancer of zeste homolog 2: from skeletal muscle differentiation to rhabdomyosarcoma carcinogenesis. *Cell Cycle* 2014, 13: 516–527
20. Palacios D, Mozzetta C, Consalvi S, Caretti G, Saccone V, Proserpio V, Marquez VE, *et al.* TNF/p38α/polycomb signaling to pax7 locus in satellite cells links inflammation to the epigenetic control of muscle regeneration. *Cell Stem Cell* 2010, 7: 455–469
21. Papait R, Cattaneo P, Kunderfranco P, Greco C, Carullo P, Guffanti A, Viganò V, *et al.* Genome-wide analysis of histone marks identifying an epigenetic signature of promoters and enhancers underlying cardiac hypertrophy. *Proc Natl Acad Sci USA* 2013, 110: 20164–20169
22. Ebinu JO, Stang SL, Teixeira C, Bottorff DA, Hooton J, Blumberg PM, Barry M, *et al.* RasGRP links T-cell receptor signaling to Ras. *Blood* 2000, 95: 3199–3203
23. Diez FR, Garrido AA, Sharma A, Luke CT, Stone JC, Dower NA, Cline JM, *et al.* RasGRP1 transgenic mice develop cutaneous squamous cell carcinomas in response to skin wounding. *Am J Pathol* 2009, 175: 392–399
24. Luke CT, Oki-Idouchi CE, Cline JM, Lorenzo PS. RasGRP1 overexpression in the epidermis of transgenic mice contributes to tumor progression during multistage skin carcinogenesis. *Cancer Res* 2007, 67: 10190–10197
25. Gbenedio OM, Bonnans C, Grun D, Wang CY, Hatch AJ, Mahoney MR, Barras D, *et al.* RasGRP1 is a potential biomarker for stratifying anti-EGFR therapy response in colorectal cancer. *JCI Insight* 2019, 4: e127552
26. Zhang X, Zhuang H, Han F, Shao X, Liu Y, Ma X, Wang Z, *et al.* Sp1-regulated transcription of RasGRP 1 promotes hepatocellular carcinoma (HCC) proliferation. *Liver Int* 2018, 38: 2006–2017
27. Hartzell C, Ksionda O, Lemmens E, Coakley K, Yang M, Dail M, Harvey RC, *et al.* Dysregulated rasgrp1 responds to cytokine receptor input in T cell leukemogenesis. *Sci Signal* 2013, 6: ra21
28. Karra L, Romero-Moya D, Ksionda O, Krush M, Gu Z, Mues M, Depeille P, *et al.* Increased baseline RASGRP1 signals enhance stem cell fitness during native hematopoiesis. *Oncogene* 2020, 39: 6920–6934
29. Li H, Gan W, Lu L, Dong X, Han X, Hu C, Yang Z, *et al.* A Genome-wide association study identifies *GRK5* and *RASGRP1* as type 2 diabetes loci in Chinese Hans. *Diabetes* 2013, 62: 291–298
30. Qu HQ, Grant SFA, Bradfield JP, Kim C, Frackelton E, Hakonarson H, Polychronakos C. Association of RASGRP1 with type 1 diabetes is revealed by combined follow-up of two genome-wide studies. *J Med Genet* 2009, 46: 553–554
31. Plagnol V, Howson JM, Smyth DJ, Walker N, Hafler JP, Wallace C, et al. Genome-wide association analysis of autoantibody positivity in type 1 diabetes cases. *PLoS Genet* 2011, 7: e1002216
32. Yasuda S, Stevens RL, Terada T, Takeda M, Hashimoto T, Fukae J, Horita T, *et al.* Defective expression of ras guanyl nucleotide-releasing protein 1 in a subset of patients with systemic lupus erythematosus. *J Immunol* 2007, 179: 4890–4900
33. Golinski ML, Vandhuick T, Derambure C, Fréret M, Lecuyer M, Guillou C, Hiron M, *et al.* Dysregulation of RasGRP1 in rheumatoid arthritis and modulation of RasGRP3 as a biomarker of TNFα inhibitors. *Arthritis Res Ther* 2015, 17: 382
34. Ksionda O, Melton AA, Bache J, Tenhagen M, Bakker J, Harvey R, Winter SS, *et al.* RasGRP1 overexpression in T-ALL increases basal nucleotide exchange on Ras rendering the Ras/PI3K/Akt pathway responsive to protumorigenic cytokines. *Oncogene* 2016, 35: 3658–3668
35. Salzer E, Cagdas D, Hons M, Mace EM, Garncarz W, Petronczki ÖY, Platzer R, *et al.* RASGRP1 deficiency causes immunodeficiency with impaired cytoskeletal dynamics. *Nat Immunol* 2016, 17: 1352–1360
36. Tan B, Wang S, Wang S, Zeng J, Hong L, Li Z, Yang J, *et al.* Genome-wide analysis of H3K27me3 in porcine embryonic muscle development. *Front Cell Dev Biol* 2021, 9: 739321
37. Wang S, Tan B, Xiao L, Zhao X, Zeng J, Hong L, Yang J, *et al.* Comprehensive analysis of long noncoding RNA modified by m6A methylation in oxidative and glycolytic skeletal muscles. *Int J Mol Sci* 2022, 23: 4600

38. Wang S, Xu X, Liu Y, Jin J, Zhu F, Bai W, Guo Y, *et al.* RIP-Seq of EZH2 identifies TCONS-00036665 as a regulator of myogenesis in pigs. *Front Cell Dev Biol* 2020, 8: 618617
39. Qiao J, Wang S, Zhou J, Tan B, Li Z, Zheng E, Cai G, *et al. ITGB6* inhibits the proliferation of porcine skeletal muscle satellite cells. *Cell Biol Int* 2022, 46: 96–105
40. Liu S, Brind'Amour J, Karimi MM, Shirane K, Bogutz A, Lefebvre L, Sasaki H, *et al. Setdb1* is required for germline development and silencing of H3K9me3-marked endogenous retroviruses in primordial germ cells. *Genes Dev* 2014, 28: 2041–2055
41. Livak KJ, Schmittgen TD. Analysis of relative gene expression data using real-time quantitative PCR and the 2–ΔΔCT method. *Methods* 2001, 25: 402–408
42. Tang Z, Li Y, Wan P, Li X, Zhao S, Liu B, Fan B, *et al.* LongSAGE analysis of skeletal muscle at three prenatal stages in Tongcheng and Landrace pigs. *Genome Biol* 2007, 8: R115
43. Zhao X, Mo D, Li A, Gong W, Xiao S, Zhang Y, et al. Comparative analyses by sequencing of transcriptomes during skeletal muscle development between pig breeds differing in muscle growth rate and fatness. *PLoS One* 2011, 6: e19774
44. Yue J, Hou X, Liu X, Wang L, Gao H, Zhao F, Shi L, *et al.* The landscape of chromatin accessibility in skeletal muscle during embryonic development in pigs. *J anim Sci Biotechnol* 2021, 12: 56
45. Ali A, Murani E, Hadlich F, Liu X, Wimmers K, Ponsuksili S. Prenatal skeletal muscle transcriptome analysis reveals novel microRNA-mRNA networks associated with intrauterine growth restriction in pigs. *Cells* 2021, 10: 1007
46. Depeille P, Henricks LM, van de Ven RAH, Lemmens E, Wang CY, Matli M, Werb Z, *et al.* RasGRP1 opposes proliferative EGFR–SOS1–Ras signals and restricts intestinal epithelial cell growth. *Nat Cell Biol* 2015, 17: 804–815
47. Myers DR, Norlin E, Vercoulen Y, Roose JP. Active tonic mTORC1 signals shape baseline translation in naive T cells. *Cell Rep* 2019, 27: 1858–1874. e6
48. Poltorak M, Meinert I, Stone JC, Schraven B, Simeoni L. S os1 regulates sustainedTCR -mediatedE rk activation. *Eur J Immunol* 2014, 44: 1535–1540
49. Iwig JS, Vercoulen Y, Das R, Barros T, Limnander A, Che Y, Pelton JG, *et al.* Structural analysis of autoinhibition in the Ras-specific exchange factor RasGRP1. *ELife* 2013, 2: e00813
50. Wu H, Huang R, Li P, Zhou L, Fu D, Li Q, et al. Association analysis between RasGRP1 gene polymorphism and body size traits of Suhuai pigs. *SWINE INDUSTRY SCIENCE* 2017, 34: 112–114
51. Roose JP, Mollenauer M, Ho M, Kurosaki T, Weiss A. Unusual interplay of two types of ras activators, RasGRP and SOS, establishes sensitive and robust ras activation in lymphocytes. *Mol Cell Biol* 2007, 27: 2732–2745
52. Zhu M, Fuller DM, Zhang W. The role of ras guanine nucleotide releasing protein 4 in FcεRI-mediated signaling, mast cell function, and T cell development. *J Biol Chem* 2012, 287: 8135–8143
53. Perez-Schindler J, Philp A. Regulation of skeletal muscle mitochondrial function by nuclear receptors: implications for health and disease. *Clin Sci* 2015, 129: 589–599
54. Zhang G, Pradhan S. Mammalian epigenetic mechanisms. *IUBMB Life* 2014, 66: 240–256
55. Wang S, Sun Y, Ren R, Xie J, Tian X, Zhao S, Li X, *et al.* H3K27me3 depletion during differentiation promotes myogenic transcription in porcine satellite cells. *Genes* 2019, 10: 231
56. Miller I, Min M, Yang C, Tian C, Gookin S, Carter D, Spencer SL. Ki67 is a graded rather than a binary marker of proliferation versus quiescence. *Cell Rep* 2018, 24: 1105–1112.e5
57. Evan GI, Vousden KH. Proliferation, cell cycle and apoptosis in cancer. *Nature* 2001, 411: 342–348
