# Supplementary material for: RASGRP1 targeted by H3K27me3 regulates myoblast proliferation and differentiation in mice and pigs : RASGRP1 regulates myoblast proliferation and differentiation
Source: Acta Biochim Biophys Sin (Shanghai). 2024 Feb 28;56(3):452–61. doi: 10.3724/abbs.2024011 (PMC10984873; doi:10.3724/abbs.2024011)
Supplement: 425FigS1-TabS1 [file 425FigS1-TabS1.pdf]

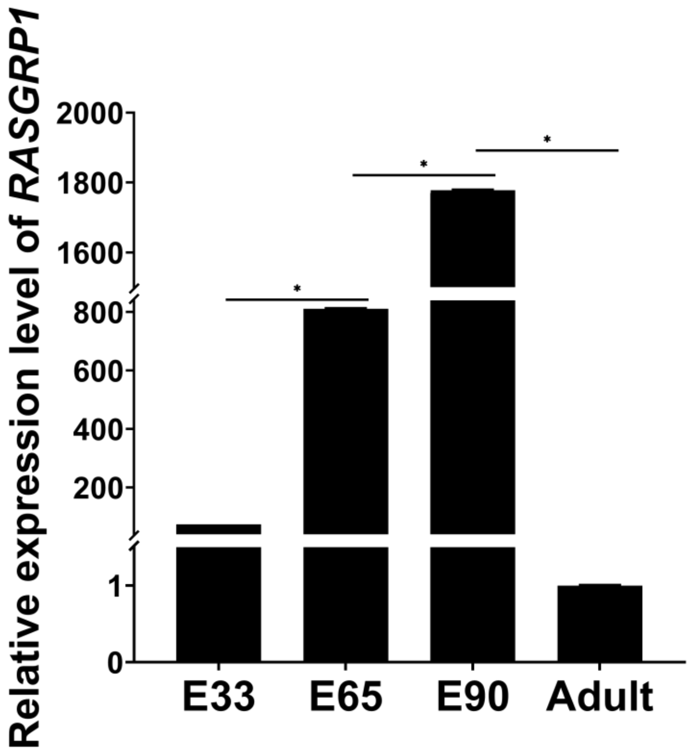

Supplementary Figure S1. The relative expression level of *RASGRP1* gene in pig skeletal muscle at E33, E65, E90 and the adult period *$P<0.05$, **$P<0.01$.

Supplementary Table S1. The sequences of the primers used in this study

Primers	Sequence (5′→3′)
Sus-*RASGRP1*-F	AGCTGGAACAGGAAATAAACACC
Sus-*RASGRP1*-R	CTAAGAACAGTCACCGTGCTC
Mus-*RASGRP1*-F	TGACAACTGTGCTGGCTTTCTCTG
Mus-*RASGRP1*-R	TTGCTGGGCTCTTGATTCGCTTC
Sus-*β-actin*-F	CCACGAGACCACCTTCAACTC
Sus-*β-actin*-R	TGATCTCCTTCTGCATCCTGT
Mus-*β-actin*-F	CTACCTCATGAAGATCCTGACC
Mus-*β-actin*-R	CACAGCTTCTCTTTGATGTCAC
Sus-*Myog*-F	AAACTACCTGCCCGTCCACCTC
Sus-*Myog*-R	GGTCCCCAGCCCCTTATCTTCC
Mus-*Myog*-F	CCATCCAGTACATTGAGCGCCTACA
Mus-*Myog*-R	ACGATGGACGTAAGGGAGTGCAGAT
Sus-*MyHC*-F	ACTGAGGAAGACCGCAAGAACATTC
Sus-*MyHC*-R	ACTTGGAGAGGTTGACGTTGGATTG
Mus-*MyHC*-F	CAAGTCATCGGTGTTTGTGG
Mus-*MyHC*-R	TGTCGTACTTGGGCGGGTTC
Sus-*Myod*-F	CGCAACGCCATCCGCTATATCG
Sus-*Myod*-R	AGTCACCGCTGTAGTGCTCTCC
Mus-*Myod*-F	CGAGCACTACAGTGGCGACTCAGAT
Mus-*Myod*-R	GCTCCACTATGCTGGACAGGCAGT
Sus-*KI67*-F	AAACCCAGATCCGAGCATATTTCTCC
Sus-*KI67*-R	CAGATTCAAACTCCACAAAGCCATAGC
Mus-*KI67*-F	ATCATTGACCGCTCCTTTAGGT
Mus-*KI67*-R	GCTCGCCTTGATGGTTCCT
Sus-*CDK1*-F	GTGGAAACCAGGAAGCCTAGCATC
Sus-*CDK1*-R	ATTCGCTTGGCAGGATCATAGACTAAC
Mus-*CDK1*-F	AGAAGGTACTTACGGTGTGGT

Mus-*CDK1*-R	GAGAGATTTCCCGAATTGCAGT
Sus-*PCNA*-F	GAGGAGGAAGCAGTTACCATAGAGATG
Sus-*PCNA*-R	ACTGAGTGTGACTGTAGGAGAGAGTG
Mus-*PCNA*-F	TTTGAGGCACGCCTGATCC
Mus-*PCNA*-R	GGAGACGTGAGACGAGTCCAT
Mus-*CDK2*-F	CCTGCTTATCAATGCAGAGGG
Mus-*CDK2*-R	GTGCTGGGTACACACTAGGTG
Mus-*CDK6*-F	GGCGTACCCACAGAAACCATA
Mus-*CDK6*-R	AGGTAAGGGCCATCTGAAAACT
Mus-*CDK12*-F	TTGGTGTCGAAGCACAAGC
Mus-*CDK12*-R	CTCCCTCCGGTCTGATTTGAA
Mus-*CDK14*-F	AAGAGCAAAATCCGTCCCTAGC
Mus-*CDK14*-R	TCATCTCAACGAAGATACAGCCA
Sus-*CDK14*-F	AGCAAGGTAAATGGGAAGTTGGTAGC
Sus-*CDK14*-R	GCAGCACTATGTTAGCATGTTTCAGTC
Mus-*CDK17*-F	TTTAAGAGGAGACTGTCCCTCAC
Mus-*CDK17*-R	TCGTTGTCCTTGCTACTGCTC
Sus-*CDK17*-F	AGCAAGGATAATGAGCCTATCGTGAAG
Sus-*CDK17*-R	ATAACGCTATGTGGTCTCCGCAAC
